# Serum lipidome analysis of healthy beagle dogs receiving different diets

**DOI:** 10.1007/s11306-019-1621-3

**Published:** 2019-12-03

**Authors:** Felicitas S. Boretti, Bo Burla, Jeremy Deuel, Liang Gao, Markus R. Wenk, Annette Liesegang, Nadja S. Sieber-Ruckstuhl

**Affiliations:** 1 Clinic for Small Animal Internal Medicine, Vetsuisse Faculty, University of Zurich, Zurich, Switzerland; 2 Singapore Lipidomics Incubator, Life Sciences Institute, National University of Singapore, Singapore, Singapore; 3 Divison of Internal Medicine, University Hospital Zurich, Zurich, Switzerland; 4 Department of Biochemistry, YLL School of Medicine, National University of Singapore, Singapore, Singapore; 5 Institute of Animal Nutrition, Vetsuisse Faculty, University of Zurich, Zurich, Switzerland

**Keywords:** Canine, Mass spectrometry, Lipid metabolism, Fatty acids, PUFA

## Abstract

**Introduction:**

Food and dietary ingredients have significant effects on metabolism and health.

**Objective:**

To evaluate whether and how different diets affected the serum lipidomic profile of dogs.

**Methods:**

Sixteen healthy beagles were fed a commercial dry diet for 3 months (control diet). After an overnight fasting period, a blood sample was taken for serum lipidomic profile analysis, and each dog was then randomly assigned to one of two groups. Group 1 was fed a commercial diet (Diet 1) and group 2 was fed a self-made, balanced diet supplemented with linseed oil and salmon oil (Diet 2) for 3 months. After an overnight fasting period, a blood sample was taken from each dog. Serum cholesterol and triacylglycerol analyses were performed and the serum lipidomic profiles were analyzed using targeted liquid chromatography–mass spectrometry.

**Results:**

Dogs fed the supplemented self-made diet (Diet 2) had significantly higher omega-3 fatty acid-containing lipids species and significantly lower saturated and mono- and di-unsaturated lipid species. Concentrations of sphingosine 1-phosphate species S1P d16:1 and S1P d17:1 were significantly increased after feeding Diet 2.

**Conclusion:**

This study found that different diets had significant effects on the dog’s serum lipidomic profile. Therefore, in studies that include lipidomic analyses, diet should be included as a confounding factor.

**Electronic supplementary material:**

The online version of this article (10.1007/s11306-019-1621-3) contains supplementary material, which is available to authorized users.

## Introduction

Lipidomics is used to identify and quantify thousands of cellular lipid molecular species and to study their interactions with other metabolites during health and disease (Burla et al. 2018a; Laaksonen et al. 2016; Pietiläinen et al. 2007; Schmitz and Ruebsaamen 2010; Yang and Han 2016). Food and dietary ingredients have important effects on metabolism and health. For example, food with an unbalanced ratio of omega-6 and omega-3 polyunsaturated fatty acids (PUFAs) seems to contribute to important health issues in humans, like atherosclerosis, obesity, and diabetes (Simopoulos 2008, 2013; Kromhout and de Goede 2014; Simopoulos 2016).

In dogs, the beneficial and detrimental effects of PUFAs are also discussed. High dietary levels of omega-3 PUFAs seem to positively influence the course of kidney disease, whereas high levels of omega-6 PUFAs cause progressive deterioration of the kidney structure (Brown et al. 1998). Furthermore, supplementation of omega-3 PUFAs were shown to improve the cachexia score in dogs with heart failure secondary to dilated cardiomyopathy, and to normalize the blood lactic acid levels leading to longer disease free intervals and survival times in dogs with lymphoma (Freeman et al. 1998; Ogilvie et al. 2000). The exact molecular basis for the dietary benefit of PUFAs is not yet completely understood and studies on the effects of PUFAs on the lipidomic profile are lacking in dogs.

Sphingolipids are a diverse class of lipids found in cell membranes (e.g., sphingomyelins) and acting as modulators of cell–cell interactions and cell recognition [e.g., sphingosine-1-phosphate (S1P)]. The signaling molecule, S1P, is involved in many biological processes such as immune reactions, vascular integrity, blood coagulation, tissue growth, and apoptosis (Thuy et al. 2014; Proia and Hla 2015; Vu et al. 2017). Alterations of S1P levels seem to occur in several metabolic disorders and may serve as prognostic and diagnostic markers in humans (Iqbal et al. 2017). In veterinary medicine as well, the S1P pathway has been suggested as a target in studying the pathogenesis of certain diseases and in developing new therapeutic interventions (Nakazawa et al. 2019; Rodriguez et al. 2015). Administration of glucocorticoids has been shown to induce S1P in dogs (Sieber-Ruckstuh et al. 2019). The various influences, however, of diets on S1P have not yet been evaluated in veterinary medicine.

Knowledge about the plasma or serum lipidome, S1P and the influence of diets and diet composition on the lipidomic profile in dogs is only sparse (Sieber-Ruckstuh et al. 2019; Lloyd et al. 2017; Yin et al. 2012). One study evaluated inter-breed signatures in the plasma lipidomic profiles of dogs (Lloyd et al. 2017). The authors found that meat type (chicken meat vs. red meat) has important effects on the plasma lipidomic profile and that much of the variance in the plasma lipidome is caused by differences in diet (Lloyd et al. 2017). A limiting factor of that study was the population of dogs, which consisted of client-owned, home-based dogs of different breeds consuming differing food types. No study has examined the effects of different diets on the serum lipidomic profiles of dogs in an experimental setting. The main objective of this study was therefore, to evaluate whether and how different diets with different protein sources, differences in fatty acid supplementation and different moisture content affect lipidomic profiles in dogs. We hypothesized that the different diets would lead to a significant change in the serum lipidomic profiles and single specific lipids. Use of an experimental setting enabled use of a standardized feeding regimen.

## Methods

### Animals and ethics statement

The study design and protocols were reviewed and approved by the Cantonal Veterinary Office of Zurich (permission number ZH213/2014). All applicable international, national, and/or institutional guidelines for the care and use of animals were followed. The study was performed using 16 purpose-bred beagles kept in groups of four dogs at the research unit of the Vetsuisse Faculty of the University of Zurich. The number of animals within one diet group was determined by performing a power analysis and by respecting the given allocation of dogs at the research unit. There were 8 intact males and 8 intact females between 4 and 6.5 years of age (median age, 4 years) and weighing between 9 and 16.3 kg (median body weight, 13.8 kg). All dogs were determined to have a body condition score (BCS) of 4/9 and considered healthy based on the results of a thorough physical examination, no observed clinical signs and no preexisting illnesses. During the experiment, physical examinations were performed at regular intervals.

### Groups and diets

At the start of the study, all 16 dogs had been fed a complete commercial dry maintenance diet twice daily (JOSIdog adult sensitive, Josera petfood GmbH & Co. KG, Kleinheubach, Germany) (Table 1) for at least 3 months. The diet consisted of dried poultry protein, rice, corn, poultry fat, beet fiber, hydrolyzed poultry protein, mineral nutrients, powdered endive root, and dried New Zealand greenshell mussel meat. Blood samples were collected from all dogs (Control diet). Dogs were then randomly assigned (equal sex distribution) to one of two groups (eight dogs in each group).

**Table 1:** Food composition of the three diets

	Control diet	Diet 1	Diet 2
Dry matter, %	97	96	72
Protein, %	25.8	32.4	39.5
Fat, %	13.4	20.1	18.3
Nitrogen-fee extracts, %	52.1	38.1	36
Ash, %	6.5	7.8	4
Crude fiber, %	2.3	1.5	2.1
ME, kcal/kg (MJ/kg) DM	3863 (16.2)	4182 (17.5)	4202 (17.6)
As fed	3748 (15.7)	4020 (16.8)	1407 (5.9)
Linoleic acid, %	3.39	2.3	0.31
Linolenic acid, %	0.29	0.1	1.28
Eicosapentaenoic acid, %	0	0.14	0.31
Docosahexaenoic acid, %	0	0.06	0.44

Food composition, expressed as percentage of food on dry matter (DM) base

Dogs of group 1 were fed a complete commercial dry maintenance diet twice daily (Diet 1; Purina Pro Plan Performance, Purina, Nestlé Purina PetCare, Vevey, Switzerland) (Table 1) to maintain ideal BCS for 3 months. The diet consisted of poultry, wheat, poultry protein, gluten, animal fat, corn, soy flour, rice, dried beet cuttings, mineral nutrients, fish oil, and corn farina.

Dogs of group 2 were fed a self-made, balanced diet enriched with linseed and salmon oil twice daily (Diet 2) to maintain ideal BCS for 3 months (Table 1). The diet consisted of beef meat, veal sternum, beef liver, carrots, broccoli, apple, fennel, vitamin E, linseed oil, and salmon oil, Kynovit Mineral, and Vitamin Optimix BARF (Yashiro et al. submitted).

### Procedures

After an overnight fast (15–16 h), a blood sample (10 mL, serum tube) was collected from each dog via jugular venipuncture. All blood samples were centrifuged after clot formation (1862×*g*, 10 min, 4 °C). The serum was removed and stored at − 80 °C until analysis.

### Clinical chemistry

Serum cholesterol and triacylglycerol parameters were analyzed at the Clinical Laboratory, Vetsuisse Faculty, University of Zurich using a Cobas Integra 800 instrument (Roche Diagnostics AG, Rotkreuz, Switzerland).

### Lipid extraction and derivatization

Lipids were extracted using a single-phase, single-step liquid–liquid extraction method with butanol:methanol as previously described (Alshehry et al. 2015; Sieber-Ruckstuh et al. 2019). Serum samples were thawed on ice and 10 µL aliquots were each mixed with 90 µL extraction mix, containing 1-butanol:methanol (1:1, v:v) and the internal standards (ISTDs): Cer d18:1/17:0 (Avanti Polar Lipids, 860517P), DG 12:0_12:0 (Avanti 800812P), LPC 20:0 (Avanti 855777P), LPE 14:0 (Avanti 856735P), PC 14:0/14:0 (Avanti 850345P), PE 14:0/14:0 (Avanti 850745P), PG 14:0/14:0 (Avanti 840445), PS 14:0/14:0 (Avanti 840033P), SM d18:1/C12:0 (Avanti 860583P), TAG 16:0_16:0_16:0 d5 (CDN Isotopes, D-5815), LacCer d18:1/16:0 d3 (Matreya LLC, 1533). All ISTDs were spiked at 50 ng/mL, except of DG, PC and TAG which spiked at 100 ng/mL into the extraction mix. After vortexing for 30 s, samples were sonicated for 30 min in an ultrasonic water bath maintained at 20 °C. Following centrifugation at 14,000*g* for 10 min (4 °C), 90 μL aliquots of the supernatants were dried under a nitrogen stream and stored at − 80 °C. For LC–MS analyses, dried extracts were reconstituted in 90 μL 1-butanol:methanol (1:1, v:v) by sonication for 10 min. A pooled Process Quality Control (PQC) sample was prepared by pooling equal volumes of each serum sample before extraction. Aliquots (10 µL) of the PQC sample were processed together with the study samples at regular intervals. Process Blank samples, which did not contain any serum, were also prepared together with the samples. For the analysis of sphingosine 1-phosphate (S1P), aliquots (50 µL) of the lipid extracts were mixed with 50 µL methanol and derivatized with 20 µL trimethylsilyldiazomethane (2 mol/L in hexanes Acros Organics, Thermo Fisher Scientific, USA). Derivatization was stopped with 1 µL glacial acetic acid (Burla et al. 2018b; Sieber-Ruckstuh et al. 2019).

### LC–MS analyses

Phospholipids, sphingolipids, and diacylglycerols were analyzed using a single-liquid chromatography–mass spectrometry (LC–MS) analysis based on published methods (Sieber-Ruckstuh et al. 2019). The LC consisted of an Agilent Zorbax RRHD Eclipse Plus C18, 2.1 × 50 mm, 1.8 µm, 95 Å column, maintained at 50 °C, and the mobile phases A: acetonitrile:water 4:6 (v:v) and B: acetonitrile:2-propanol 1:9 (v:v); both mobile phases contained 10 mmol/L ammonium formate. An Agilent 1290 (G4220A) Infinity Binary pump was used for an LC gradient consisting of 0–2 min: 20% B, 2–7 min: 20% to 60% B, 7–9 min: 60% to 100% B, 9–9.1 min: 100% to 20% B, 9.1–10.8 min: 20% B. The flow rate was 0.4 mL/min and the sample injection volume was 0.5 µL. The MS consisted of an Agilent 6495 triple quadrupole MS run in positive mode with the source parameters of gas temperature: 200 °C, gas flow: 12 L/min, nebulizer: 25 psi, sheath gas heater: 250 °C, sheath gas flow: 12 L/min, capillary voltage: 3500 V, Vcharging: 500. The ion funnel parameters were positive high pressure: RF 150, positive low pressure: RF 60.

The triacylglycerol species were measured using a separate method and the same column and mobile phases as the LC–MS method describe above, but a different gradient was used: 0–1 min: 10% to 70% B, 1–10 min: 70% to 100% B, 10–12 min: 100% B, 12–12.1 min: 100% to 10% B, 12.1–14 min: 10% B. The flow rate was 0.4 mL/min and the sample injection volume was 2 µL. An Agilent 6490 triple quadrupole MS was used in positive mode with the source parameters of gas temperature: 250 °C, gas flow: 14 L/min, nebulizer: 35 psi, sheath gas heater: 250 °C, sheath gas flow: 11 L/min, capillary voltage: 3500 V, Vcharging: 1000. The ion funnel parameters were positive high pressure: RF 150, positive low pressure: RF 60. For both analyses, the data were collected in dynamic multiple reaction monitoring mode. The results for the transitions and collision energies are presented in Supplementary EXCEL Tables S1 and S2, respectively. The sphingosine 1-phosphate species were also measured using LC–MS, as previously described (Narayanaswamy et al. 2014; Sieber-Ruckstuh et al. 2019). Peak identification, integration, normalization, and quality control filtering were performed as described by Sieber-Ruckstuh et al. (2019). Lipid species with a median peak area in the PQC less than 5 times of the Process Blank samples or below an absolute area of 200 were discarded. Furthermore, only lipid species with an analytical coefficient of variation (CV) below 25%, based on normalized peak areas in PQC samples, were considered for downstream data analysis. Relative abundances of lipid species were calculated based on normalization with ISTDs (Supplementary Table S1), which were spiked-in at known concentrations. When normalization lead to an increase of analytical CV, the average ISTD peak area in the PQC samples was used for normalization and quantification of species from the corresponding class (see Supplementary Table S1). Calculated relative abundances of lipid species (Supplementary EXCEL Table S3) correspond to estimated concentrations and are expressed as μmol/L (Burla et al. 2018a). The results for normalization are presented in Supplemental Information Table S1). The multiple reaction monitoring raw data were processed using Agilent Masshunter Quantitative Analysis software (B.08), the data post-processing was performed using R version 3.6.0 (R Core Team 2017).

### Statistics and data visualization

The statistical significance of changes in measured serum cholesterol and triacylglycerol concentrations and lipid abundances within a group (Diet 1 vs. Control diet; Diet 2 vs. Control diet) were determined using paired, two-tailed *t*-tests from log2-transformed values. Differences between groups (Diet 2 vs. Diet 1) were determined using unpaired, two-tailed Welch’s *t*-tests from log2-transformed values. False discovery rate (FDR)-adjusted *P* values were calculated using the Benjamini–Hochberg procedure (Benjamini and Hochberg 1995). The R packages FactoMineR and factoextra (Husson et al. 2017; Kassambara and Mundt 2017) were used to create the principal component analysis (PCA) plot from scaled, centered log2-transformed lipid abundance values. Figures for cholesterol, triacylglycerol, and single lipid species were generated and statistically analyzed using GraphPad Prism version 6.

## Results and discussion

This study found that different diets significantly influence the serum lipidomic profile in beagle dogs. Use of an experimental study design allows standardization of dietary intake, which can be challenging when a field study is used. In an experimental setting, the food composition can be exactly designed (e.g., red meat, white meat, fish, PUFA supplementation) and the dogs can be exclusively fed this standardized diet over a long period. Therefore, dogs could represent an important large animal model to study lipid metabolism and the effects of diets and diet compositions on health and disease.

### Health of the dogs

In none of the dogs, did diet modification lead to a change in appetite or to any adverse events during the study. Food rations were adapted to maintain stable body weights and BCSs. All intact female dogs were out of season during the study. This is an important aspect, as changes in hormonal status in female dogs could potentially alter food intake or lipid metabolism.

### Diet composition

Comparing the composition of the three diets revealed that Diet 1 had the highest crude fat content, followed by Diet 2 and Control diet. For crude protein, nitrogen-free extract, crude ash, and crude fiber, the diets ranked as Diet 2 > Diet 1 > Control diet, Control diet > Diet 1 > Diet 2, Diet 1 > Control diet > Diet 2, and Control diet > Diet 2 > Diet 1, respectively (Table 1). Diet 2 was a home-made, balanced raw-food diet (RFD). RFDs typically consist of approximately 70–90% meat, giblets, and bones, and 10–30% vegetables and fruits. Composition varies depending on the developer (e.g., Londsdale, Billinghurst, Simon) of the diet and the origin of the recipe (e.g., book, internet, breeders, and friends) (Becker et al. 2012). Due to the high concentrations of (70–90%) of meat, giblets, and bones, RFDs often contain higher protein and fat concentrations and lower carbohydrate and fiber concentrations, compared with commercial dry food diets (Freeman et al. 2013). The RFD of this study had the highest protein concentration, but not the lowest fiber concentration, compared to the two commercial diets.

### Serum cholesterol and triacylglycerol concentrations

**Fig. 1:**
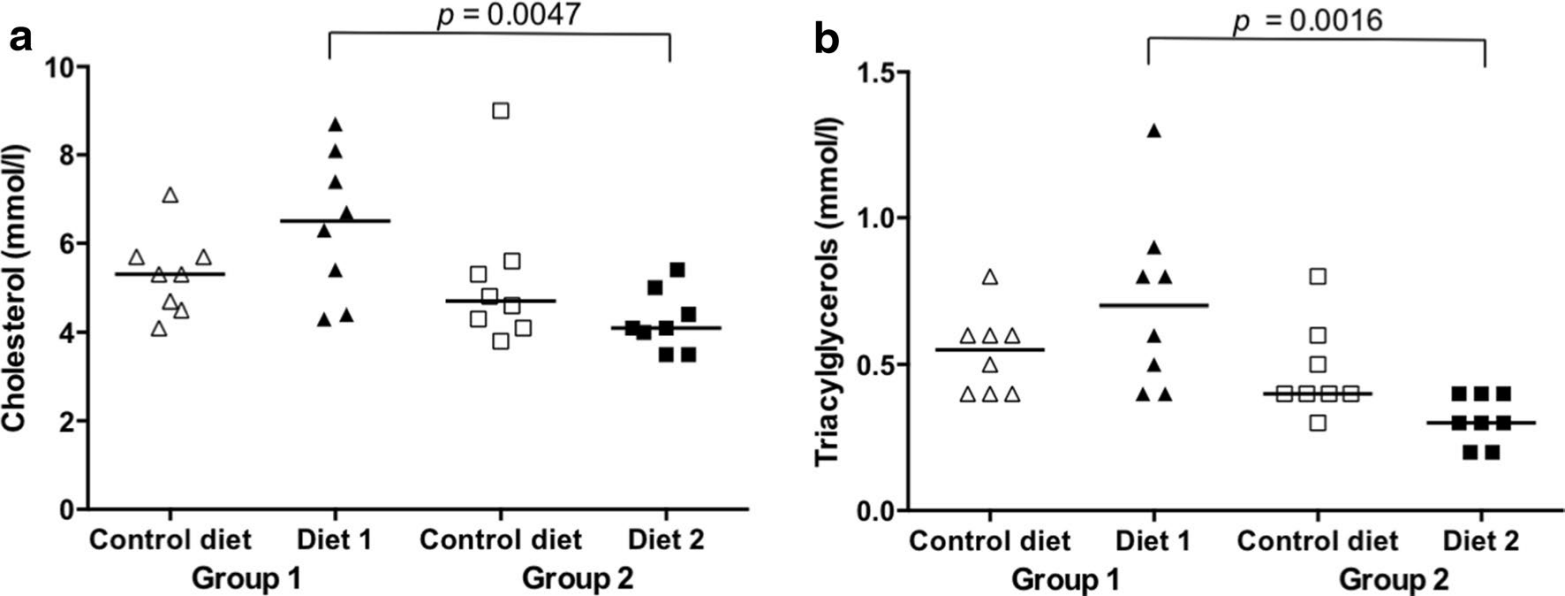
**a**, **b** Changes in serum cholesterol and triacylglycerol concentrations. Levels for each dog of group 1 (triangles) and group 2 (squares). Control diet (open symbols), levels before diet change. Diet 1 and Diet 2 (closed symbols), levels after 3 months of feeding Diet 1 or Diet 2, respectively

Feeding Diet 1 and Diet 2 did not reveal significant changes in cholesterol or triacylglycerol concentrations compared to feeding Control diet (Fig. 1). Dogs fed Diet 1, however, had significantly higher cholesterol and triacylglycerol concentrations than dogs fed Diet 2 (Fig. 1). The crude fat content was highest in Diet 1 followed by Diet 2 and lowest in the Control diet (Table 1). Therefore, it seems not surprising that dogs fed Diet 1 had the highest cholesterol and triacylglycerol concentrations. However, even though the Control diet had the lowest crude fat content, the cholesterol and triacylglycerol concentrations of the dogs fed the Control diet were not the lowest. One reason could be the linseed oil supplementation used in Diet 2. Linseed oil is a source of plant sterols (Schwartz et al. 2008), which has been described to reduce total cholesterol and low-density lipoprotein cholesterol in humans (Doornboos et al. 2006; Szymańska et al. 2012). Plant sterols inhibit both the absorption of dietary cholesterol and the reabsorption of cholesterol from the bile (Piironen et al. 2000). Among the vegetable oils, linseed oil contains the highest level of the omega-3 fatty acid alpha-linolenic acid (ALA, 18:3 (*n *− 3)) (Matthäus and Musazcan Özcan 2015). Dietary supplementation with ALA results in a significant decrease in triacylglycerol and low-density lipoprotein cholesterol concentrations in humans, which is accompanied by a reduction in the low- to high-density lipoprotein ratio (Dittrich et al. 2015). Therefore, the addition of linseed oil to Diet 2 likely affected the cholesterol and triacylglycerol concentrations in this treatment group.

### Changes of the overall serum lipidomic profile

**Fig. 2:**
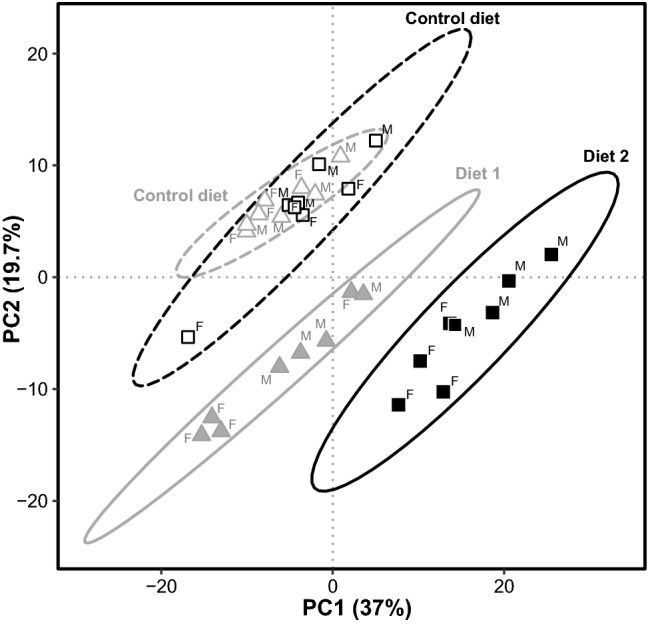
Principal component analysis (PCA) of the serum lipidomic data set showing the serum samples of dogs of group 1 (triangles) and group 2 (squares). Control diet (open symbols), levels before diet change. Diet 1 and Diet 2 (closed symbols), levels after 3 months of feeding Diet 1 or Diet 2, respectively. Female dogs are marked as F and male dogs as M

The PCA revealed that the abundances of the measured lipid species separated the samples of groups 1 (Diet 1) and group 2 (Diet 2) and showed the distinct effects of the different diets (Fig. 2). One dog fed the Control diet was an outlier compared with the other dogs on the Control diet. This was an intact female dog. Clinically the dog was unremarkable, had normal appetite and did not show any obvious clinical signs of heat. The total cholesterol concentration, however, was increased (9.0 mmol/L, other dogs: 3.8–7.1) compared to the other dogs. This difference most likely explains why this dog was so different from the other dogs fed the Control diet in the PCA plot.

### Alteration of serum lipids containing specific fatty acids

Volcano plots comparing diets revealed significant changes in serum concentrations of many lipid species (Supplemental Information Figs. S1–S6). After feeding Diet 2, levels of most of the triacylglycerol (TG) species were significantly lower than during feeding Control diet or Diet 1. Again, supplementation of the Diet 2 with linseed oil seems the most likely explanation for this difference. In contrast, many lipid species containing linolenic acid and eicosapentaenoic acid (EPA) and lipid species that contained omega-3 PUFAs (e.g., PC 36:5) were up-regulated. Only the sum compositions (total chain length and the total number of unsaturations) of phospholipids were determined in this study given the employed analytical method (Liebisch et al. 2013). These sum compositions comprise lipid species with different FA compositions (Zacek et al. 2016). These findings will be discussed in more detail below.

**Fig. 3:**
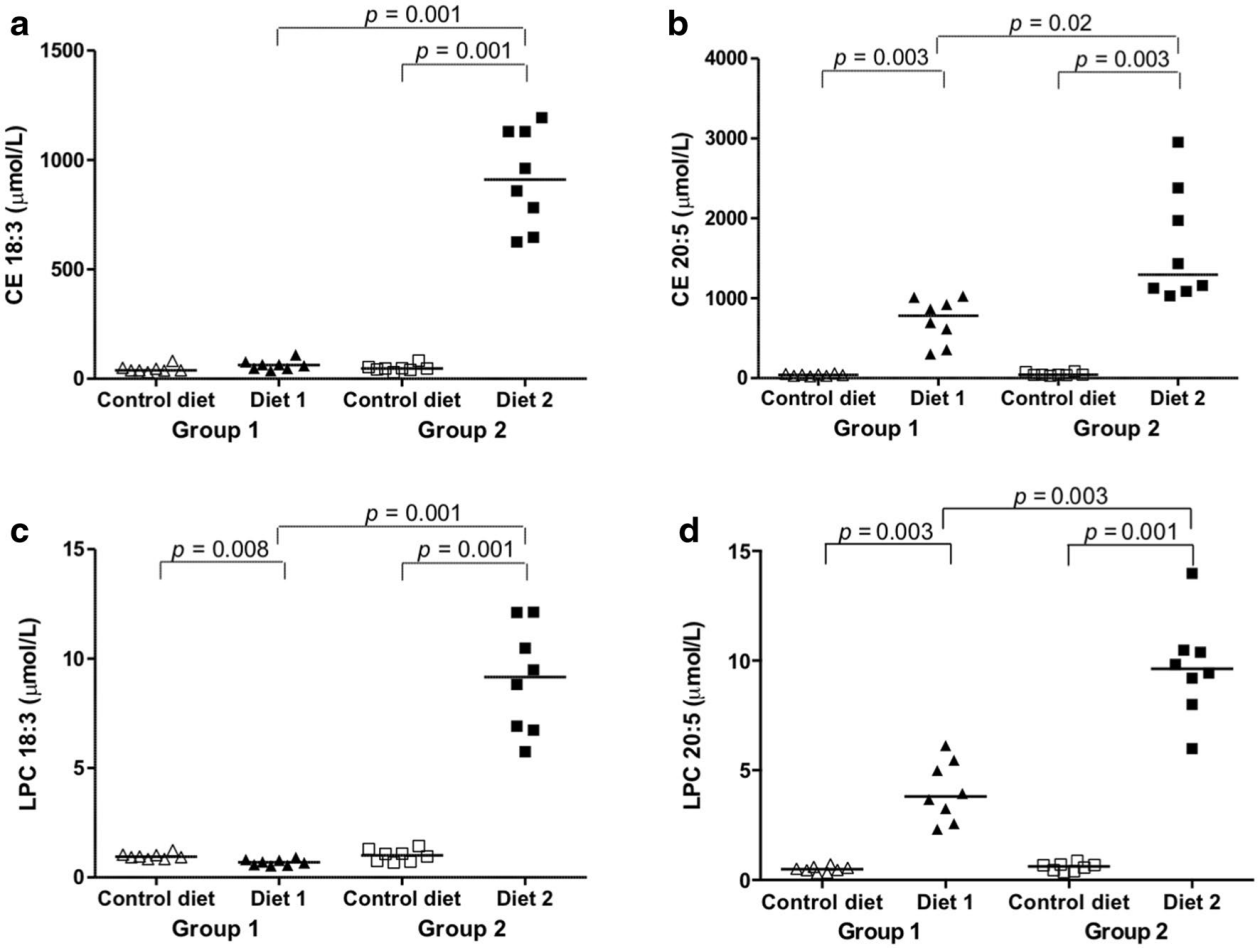
**a**–**d** Changes in single lipid species. Levels for each dog of group 1 (triangles) and group 2 (squares). Control diet (open symbols), levels before diet change. Diet 1 and Diet 2 (closed symbols), levels after 3 months of feeding Diet 1 or Diet 2, respectively. CE, cholesteryl ester; LPC, lysophosphatidylcholine

The concentration of linolenic acid-containing lysophosphatidylcholine (LPC 18:3) was significantly decreased, and that of EPA-containing cholesteryl ester (CE 20:5) and LPC 20:5 were significantly increased after feeding Diet 1 than during feeding Control diet. The concentrations of linolenic acid-containing CE 18:3, LPC 18:3, and of EPA-containing CE 20:5 and LPC 20:5 were significantly increased after feeding Diet 2, compared to feeding Control diet (Fig. 3). This resulted in increased concentrations of all four EPA- and linolenic acid-containing lipid species in dogs fed Diet 2 compared to dogs fed Diet 1 (Fig. 3, Supplementary EXCEL Tables S3 and S4, Supplementary Information Fig. S7). Comparing the linolenic acid and EPA contents of the diets revealed that Diet 2 contained the highest amounts of linolenic acid and EPA; Diet 1 had a higher EPA content than Control diet. The increased serum levels of these lipid species can therefore be explained by their increased levels in the diets they received. Diet 2 was supplemented with salmon oil and linseed oil. Fish with a high fat content and fish oils have high concentrations of long chain omega-3 FAs, mostly EPA and docosahexaenoic acid (Brown et al. 1991). Terrestrial plants (e.g., linseed) provide only ALA (Venegas-Calerón et al. 2010). Supplementation of the canine diets with these oils had direct effects on serum levels of lipid species containing omega-3 fatty acids. This is consistent with gas chromatographic results of studies that found that supplementation of dog diets with omega-3 PUFAs affects FA profiles (Wander et al. 1997; Hall et al. 2006; Stoeckel et al. 2011).

**Fig. 4:**
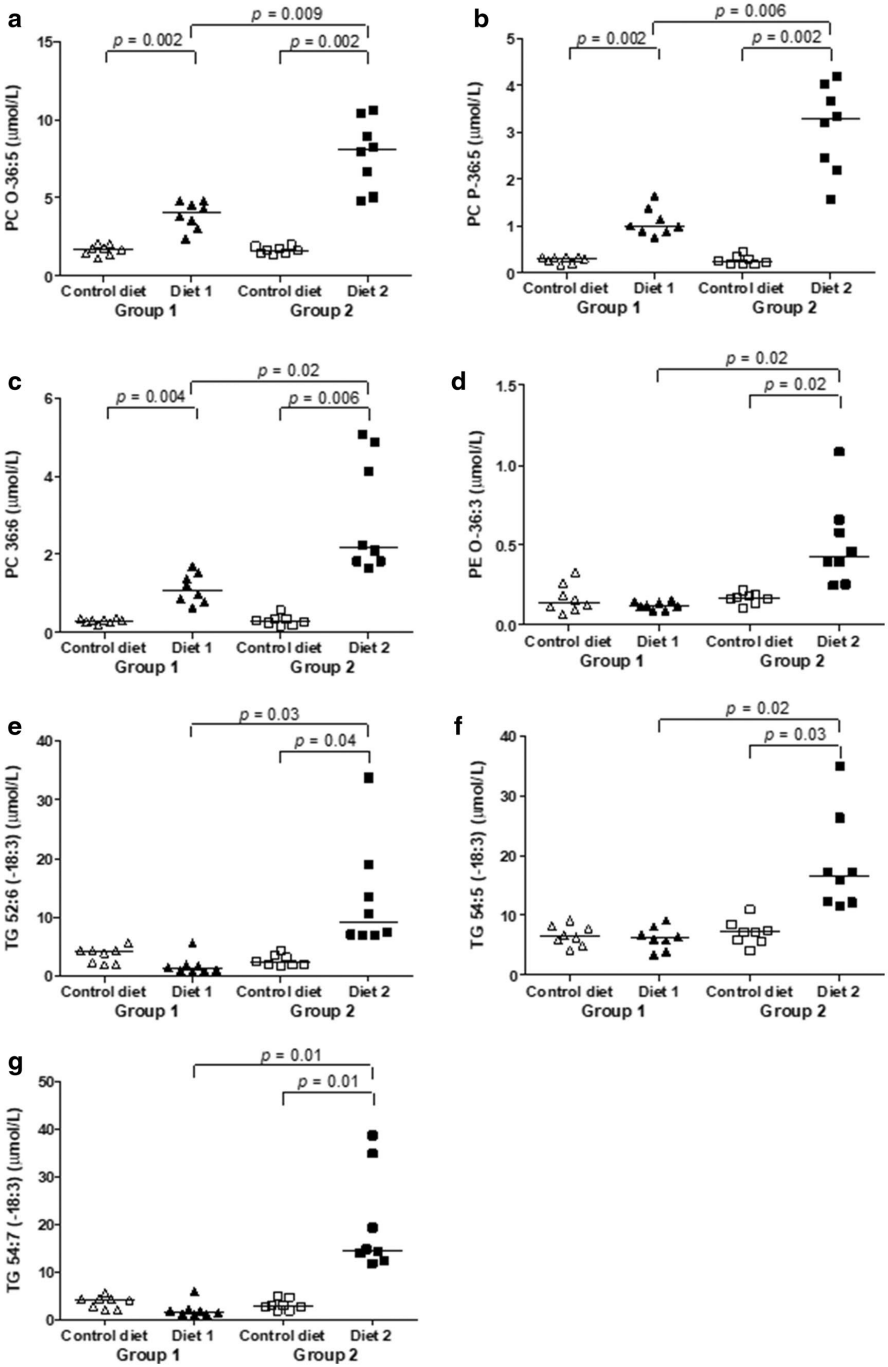
**a**–**g** Changes in single lipid species. Levels for each dog of group 1 (triangles) and group 2 (squares). Control diet (open symbols), levels before diet change. Diet 1 and Diet 2 (closed symbols), levels after 3 months of feeding Diet 1 or Diet 2, respectively. *PC-O* ether-phosphatidylcholine, *PC-P* plasmalogen phosphatidylcholine, *PC* phosphatidylcholine, *PE-O* ether-phosphatidylethanolamine, *TG* triacylglycerol

The ether-phosphatidylcholine (PC-O) 36:5, the plasmalogen phosphatidylcholine (PC-P) 36:5, and the phosphatidylcholine (PC) 36:6 concentrations were significantly higher after feeding Diet 1 than during feeding Control diet (Fig. 4). PC O-36:5, PC P-36:5, PC 36:6, ether-phosphatidylethanolamine (PE-O) 36:3, TG 52:6 [−18:3], TG 54:5 [−18:3], and TG 54:7 [−18:3] concentrations were all significantly increased feeding Diet 2, compared to feeding Control diet (Fig. 4). Finally, all lipid species were significantly higher in dogs fed Diet 2 compared to dogs fed Diet 1 (Fig. 4). All these lipids species contain or potentially consist of species that contain linolenic acid or EPA. Therefore, the increased concentrations of linolenic acid- and EPA-containing lipids in the dogs fed Diet 2 was an expected result. Due to supplementation with linseed and salmon oil, this diet had the highest amounts of linoleic acid and EPA. Also, as above, the increased concentrations of PC O-36:5, PC P-36:5, and PC 36:6 after feeding Diet 1 compared to feeding Control diet can be explained by the presence of higher amounts of EPA in Diet 1.

**Fig. 5:**
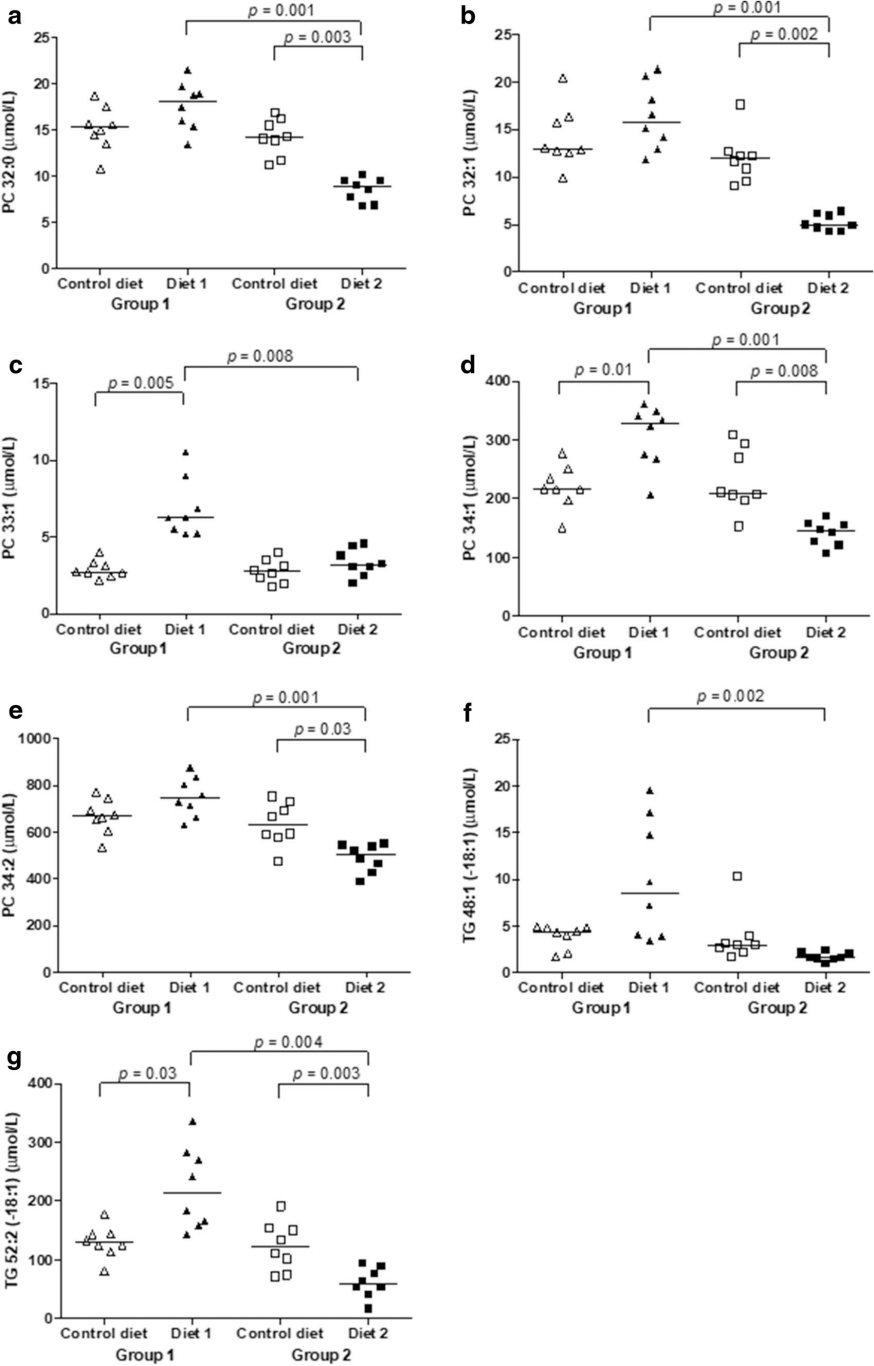
**a**–**g** Changes in single lipid species. Levels for each dog of group 1 (triangles) and group 2 (squares). Control diet (open symbols), levels before diet change. Diet 1 and Diet 2 (closed symbols), levels after 3 months of feeding Diet 1 or Diet 2, respectively. *PC* phosphatidylcholine, *TG* triacylglycerol

The saturated, mono- and di-unsaturated lipid species, PC 33:1, PC 34:1 and TG 52:2 [−18:1] were significantly higher after feeding Diet 1 than during feeding Control diet (Fig. 5). The saturated, mono- and di-unsaturated lipid species, PC 32:0, PC 32:1, PC 34:1, PC 34:2, and TG 52:2 [−18:1] were significantly lower after feeding Diet 2 than during feeding Control diet (Fig. 5). This resulted in significantly higher PC 32:0, PC 32:1, PC 33:1, PC 34:1, PC 34:2, TG 48:1 [−18:1] and TG 52:2 [−18:1] concentrations in dogs fed Diet 1, compared to dogs fed Diet 2. Diet 2 contained beef as the main meat component; the Control diet and the Diet 1 contained poultry meat. White meat (e.g., poultry) and fish have lower total amounts of saturated and monounsaturated fats compared to red meat (e.g., beef) (Williams 2007). Consumption of a diet high in red meat results in increased plasma total saturated and monounsaturated fatty acid and triacylglycerol concentrations (Lloyd et al. 2017; Wolmarans et al. 1991). Therefore, the lower concentrations of saturated, mono- and di-unsaturated lipid species in dogs fed Diet 2 was surprising. However, the fatty acid content of meat in different regions of the world varies. Beef from pasture-fed cattle is usually higher in polyunsaturated fatty acids, compared with beef from cattle fed grain (Williams 2007). In Switzerland, dairy cows are commonly fed pasture-based diets. The main dietary components are therefore grass-based, which results in high amounts of unsaturated fatty acids in the milk and possibly in the meat (Baars et al. 2019; Srednicka-Tober et al. 2016). The differences in saturated and monounsaturated fat content between beef and chicken meat in Switzerland is likely not as high as in other countries (e.g., USA) where cattle are mainly fed grain. Another explanation for the decreased abundance of saturated and monounsaturated FAs in dogs fed Diet 2 is, again, the dietary supplementation with linseed and salmon oil. As discussed above, supplementing a diet with these omega-3 fatty acid leads to significant increases in PUFAs. Concurrently, however, omega-3 supplementation significantly reduces (although to a lesser extent) the plasma concentrations of saturated and monounsaturated fats (Hall et al. 2006).

**Fig. 6:**
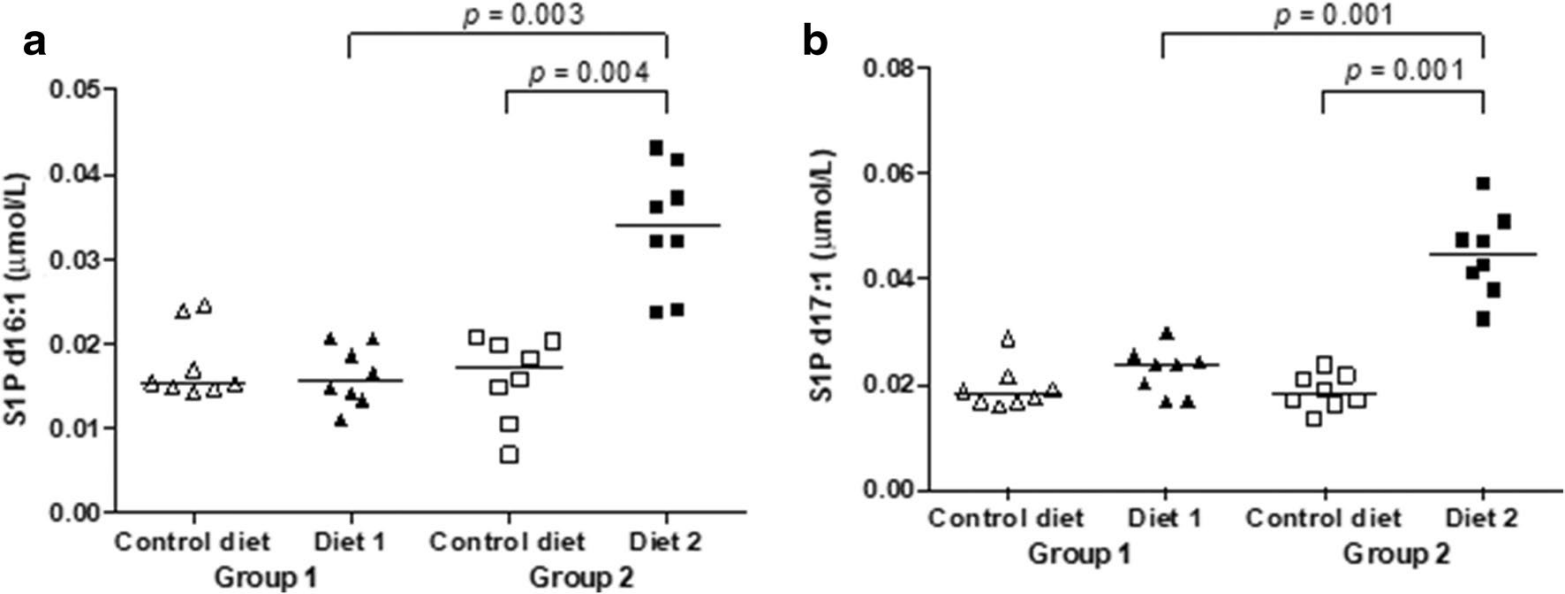
**a**, **b** Changes in single lipid species. Levels for each dog of group 1 (triangles) and group 2 (squares). Control diet (open symbols), levels before diet change. Diet 1 and Diet 2 (closed symbols), levels after 3 months of feeding Diet 1 or Diet 2, respectively. *S1P* sphingosine 1-phosphate

The concentrations of S1P species d16:1 and S1P d17:1 significantly increased after feeding Diet 2. The values were also significantly higher in dogs fed Diet 2 compared to dogs fed Diet 1 (Fig. 6). S1P is a sphingolipid belonging to a class of important structural and signaling lipids. In a previous study we could show that short-term treatment of healthy dogs with prednisolone led to a significant increase of the major plasma S1P species (e.g., S1P d16:1, S1P d18:0, S1P d18:1, S1P d18:2) (Sieber-Ruckstuh et al. 2019). The specific functions of the S1P d16:1 and 17:1 species are so far unknown. However, the d16:0- and d16:1-containing sphingolipids seem to have different biophysical and biological properties than the more abundant d18-containing sphingolipids and seem to be involved in cardiac function (Russo et al. 2013). In mice, a diet high in saturated fats resulted in increased concentrations of d16-containing sphingolipids (Russo et al. 2013). Further studies are needed to better understand the physiology and pathophysiology of S1P.

This study has some limitations. First, no cross-over study design was used and the diets themselves were not analyzed with the targeted lipidomics approach. This would have helped to better clarify the effects of the different ingredients of the diets on the lipid profile and to exclude intraindividual effects. We used triple tandem quadrupole MS operated only in positive ion mode for the analyses. The limitation of this approach is that the positive ion mode does not give any information about the fatty acid composition of phospholipids (Hsu and Turk 2009). Finally, biological variation in the study population could also be a limitation; the animals used were different sexes and ages. Only beagle dogs were included, which reduced the variance in breed. However, study results suggest that the beagle breed exhibits high inbreed variance in urine metabolites and in the plasma lipidome (Beckmann et al. 2010; Lloyd et al. 2017).

In conclusion, this study was the first to describe targeted lipidomic analysis of the serum of dogs fed different diets with different protein sources, different fatty acid supplementation and different moisture content in an experimental setting. Dietary omega-3 FA supplementation resulted in a significant increase in omega-3 FA-containing lipid concentrations and a significant decrease in unsaturated and monounsaturated fat concentrations. The study found that diet composition (e.g., fat content and source, PUFA supplementation) significantly affects the blood lipidomic profile. Diet composition should therefore be considered during interpretation of lipidomic data from in vivo conditions. During experimental studies, diet composition should whenever possible be standardized and left unchanged to increase the accuracy and validity of the results.

## Electronic supplementary material

Below is the link to the electronic supplementary material.

Supplementary material 1 (XLSX 257 kb)

Supplementary material 2 (DOCX 8704 kb)

## Data Availability

We provide the full lipidomics dataset and statistical results for each lipid species in the supplementary information. The Supplementary Information includes Supplementary EXCEL Tables S1–S4 and Supplementary Information including one Supplementary Table S1 and seven Supplementary Figs. S1–S7. All mass spectrometry raw data files, unprocessed data files and R scripts used for the data analysis and figures can be obtained from the authors at any time.

## Abbreviations

AA: Arachidonic acid
ALA: Alpha-linolenic acid
BCS: Body condition score
CE: Cholesteryl ester
DHA: Docosahexaenoic acid
EPA: Eicosapentaenoic acid
FA(s): Fatty acid(s)
FDR: False discovery rate
HDL: High-density lipoprotein
LC–MS: Targeted liquid chromatography–mass spectrometry
LDL: Low-density lipoprotein
LPC: Lysophosphatidylcholine
MRM: Multiple reaction monitoring
PC: Phosphatidylcholine
PCA: Principal component analysis
PC-O: Ether-phosphatidylcholine
PC-P: Plasmalogen phosphatidylcholine
PE-O: Ether-phosphatidylethanolamine
PQC: Process quality control
PUFAs: Polyunsaturated fatty acids
RFD: Raw-food diet
S1P: Sphingosine 1-phosphate
TG: Triacylglycerol
