# Supplementary material for: Serum lipidome analysis of healthy beagle dogs receiving different diets
Source: Metabolomics. 2019 Dec 3;16(1):1. doi: 10.1007/s11306-019-1621-3 (PMC6890591; doi:10.1007/s11306-019-1621-3)

**Supplementary Information**

**Serum lipidome analysis of healthy beagle dogs receiving different diets**

Felicitas S. Boretti^1^, Bo Burla^2^, Jeremy Deuel^3^, Liang Gao^2^, Markus R. Wenk^2,4^, Annette Liesegang^5^, Nadja S. Sieber-Ruckstuhl^1^

^1^Clinic for Small Animal Internal Medicine^*^, Vetsuisse Faculty, University of Zurich Switzerland

^2^Singapore Lipidomics Incubator, Life Sciences Institute, National University of Singapore, Singapore

^3^Divison of Internal Medicine, University Hospital Zurich, Switzerland
^4^Department of Biochemistry, YLL School of Medicine, National University of Singapore

^5^Institute of Animal Nutrition, Vetsuisse Faculty, University of Zurich, Switzerland

**Corresponding authors:**

Annette Liesegang

Institute of Animal Nutrition, Vetsuisse Faculty, University of Zurich, Switzerland

[aliese@nutrivet.uzh.ch](mailto:aliese@nutrivet.uzh.ch), phone: +41 44 635 83 01, Fax: +41 44 635 89 20 and

Nadja Sieber-Ruckstuhl

Clinic for Small Animal Internal Medicine, Vetsuisse Faculty, University of Zurich Switzerland, [nsieber@vetclinics.uzh.ch](mailto:nsieber@vetclinics.uzh.ch), phone: +41 44 635 83 01, Fax: +41 44 635 81 11

**ORCID number of authors:**

FSB 0000-0001-6793-8464 ; BB 0000-0002-5918-3249; JD 0000-0002-5409-7712; LG 0000-0003-3700-1069; MRK 0000-0001-5447-7881; AL 0000-0002-4292-8515; NSSR 0000-0002-8256-0137

**Running title**: Effects of feeding on serum lipidome

**Supplementary Table S1: Internal standards and methods used to normalize and calculate relative abundances of species from different lipid classes.** Spiked-in internal standards (ISTD, second column) used to normalize the raw peak areas and to quantify the relative abundances of all species from the corresponding lipid class (first column). The normalization and quantification method varied between different lipid classes and are detailed under the column Remarks. No symbol indicates that the peak area of indicated ISTD was used to normalize and quantitate species from the lipid class in each sample. (1) indicates that the average peak area of the ISTD in all Process Quality Control (PQC) samples was used to normalize and quantify the relative abundances of all species from the corresponding lipid class in each sample. (2) indicates that an ISTD from another lipid class was used for normalization and quantification of all species from the lipid class in each sample. (3) indicates that the abundance was further corrected with an in-house determined method/instrument specific response factor. See Methods for more details.

| Lipid Class | Internal Standard | Remarks |
| --- | --- | --- |
| CE | TAG 48:0 d5 (IS) | 1, 2, 3 |
| Cer | Cer d18:1/17:0 (IS) | 1 |
| DG | TAG 48:0 d5 (IS) | 1, 2, 3 |
| GM3 | Cer d18:1/17:0 (IS) | 1, 2 |
| HexCer | Cer d18:1/17:0 (IS) | 1, 2 |
| Hex2Cer | Cer d18:1/17:0 (IS) | 1, 2 |
| LPC | LPC 20:0 (IS) | 1 |
| LPC-O | LPC 20:0 (IS) | 1, 2 |
| LPE | LPE 14:0 (IS) | 1 |
| LPI | PC 28:0 (IS) | 1, 2, 3 |
| PC | PC 28:0 (IS) |  |
| PC-O | PC 28:0 (IS) | 2 |
| PC-P | PC 28:0 (IS) | 2 |
| PE | PE 28:0 (IS) | 1 |
| PE-O | PE 28:0 (IS) | 1, 2 |
| PE-P | PE 28:0 (IS) | 1, 2 |
| PG | PG 28:0 (IS) |  |
| PI | PC 28:0 (IS) | 1, 2, 3 |
| PS | PS 28:0 (IS) |  |
| S1P | S1P d18:1-13C2D2 (IS) |  |
| SM | SM 30:1 (IS) |  |
| TAG | TAG 48:0 d5 (IS) | 1 |

**Supplementary EXCEL Table S1-S2:** Lists of monitored MRM transitions with collision energies used in the phospholipid/sphingolipid/diacylglycerol and the triacylglycerol methods, respectively.

**Supplementary EXCEL Table S3:** Relative concentrations of measured lipid species in all measured serum samples. Relative concentrations correspond to estimated concentrations expressed as µmol/L and were calculated based on the normalization and quantification methods described in Methods and Supplementary Information Table S1.

**Supplementary EXCEL Table S4:** Average log2-fold changes (log2FC), fold changes (FC), *P* values (pValue) and FDR-adjusted *P* values (FDR-pValue) from comparisons of different diet groups for the levels of all quantified serum lipid species. Test results are from paired, two-tailed *t*-tests in case of Diet 1 *vs* Control diet and Diet 2 *vs* Control diet, and from unpaired, two-tailed Welch’s *t*-tests in case of Diet 2 *vs* Diet 1.

**Supplementary FigS1:** Volcano plot comparing triacylglycerol (TG) species between Diet 1 and Control diet. Significantly changed TG species (FDR-adjusted p value < 0.05, |FC| > 1.5, paired, two-tailed *t*-test) are indicated in red with the species name. A positive FC means species has higher serum level in dogs fed Diet 1 compared to dogs fed Control diet.

**
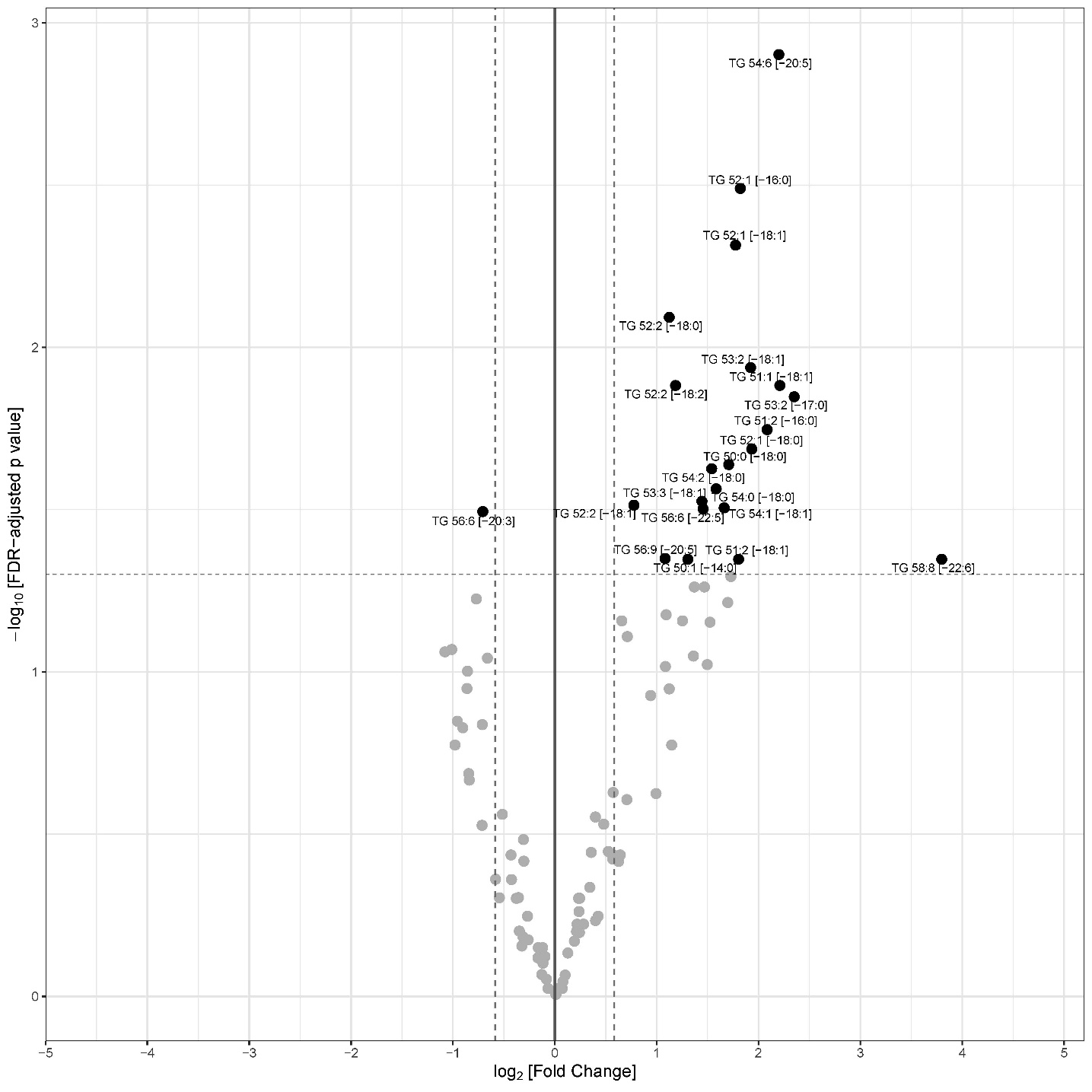
**

**Supplementary FigS2:** Volcano plot comparing triacylglycerol (TG) species between Diet 2 and Control diet. Significantly changed TG species (FDR-adjusted p value < 0.05, |FC| > 1.5, paired, two-tailed *t*-test) are indicated in red with the species name. A positive FC means species has higher serum level in dogs fed Diet 2 compared to dogs fed Control diet.

**
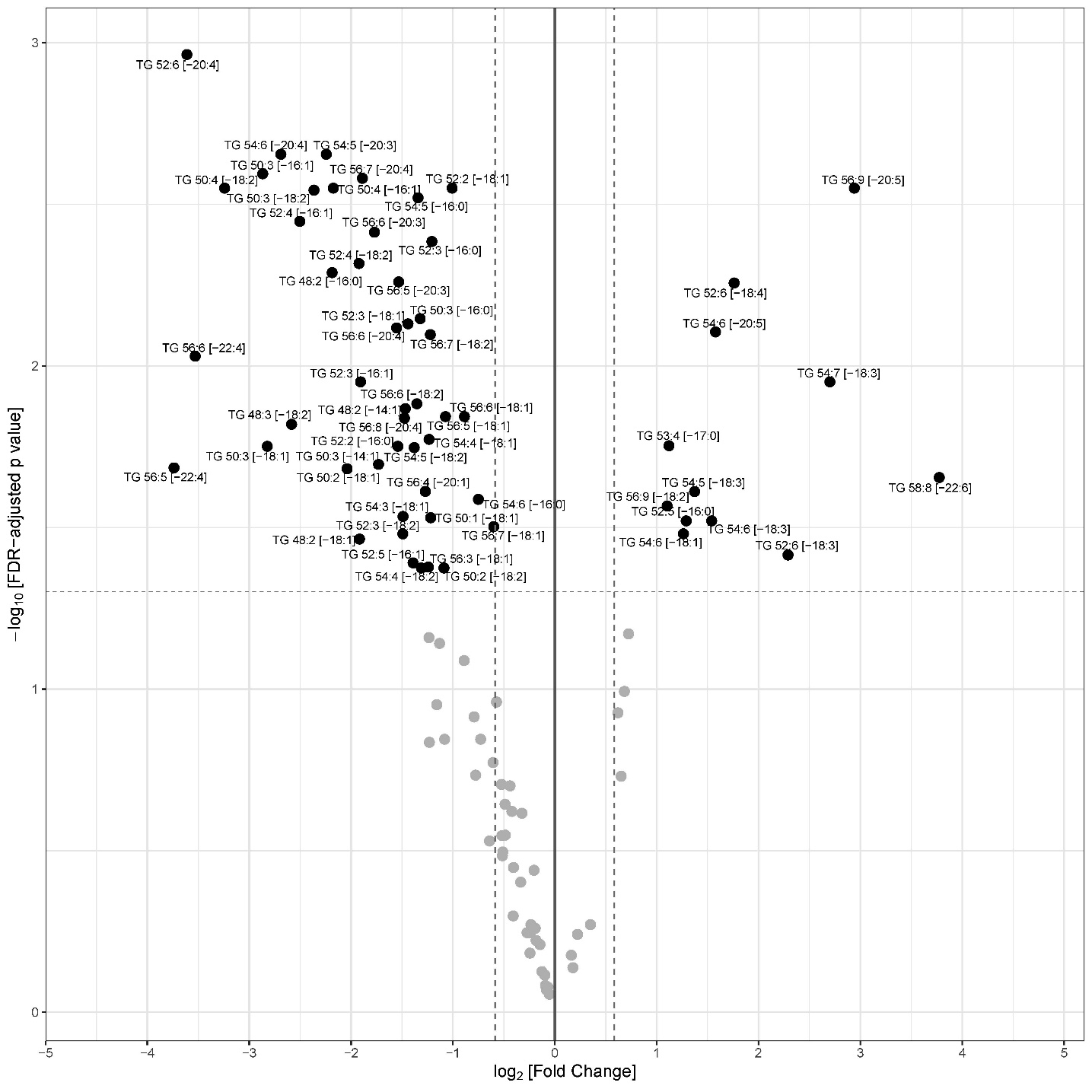
**

**Supplementary FigS3:** Volcano plot comparing triacylglycerol (TG) species between Diet 2 and Diet 1. Significantly changed TG species (FDR-adjusted p value < 0.05, |FC| > 1.5, unpaired, two-tailed Welch’s *t*-test) are indicated in red with the species name. A positive FC means species has higher serum level in dogs fed Diet 2 compared to dogs fed Diet 1.

**
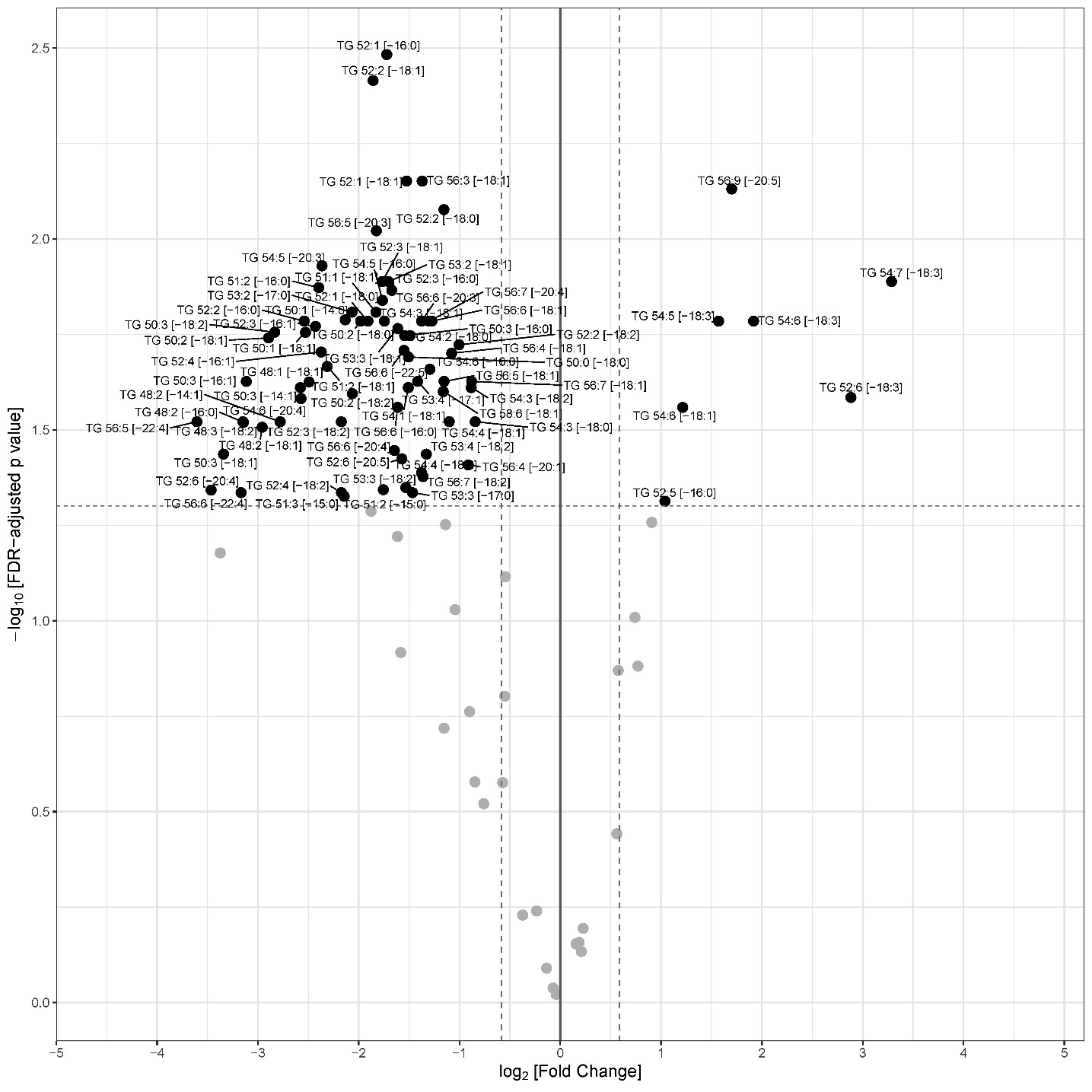
**

**Supplementary FigS4:** Volcano plot comparing all quantified lipid species, except of TGs, between Diet 1 and Control diet. Significantly changed lipid species (FDR-adjusted p value < 0.05, |FC| > 1.5, paired, two-tailed *t*-test) are indicated in red with the species name. The corresponding plot for TGs are shown in Supplementary FigS1. A positive FC means species has higher serum level in dogs fed Diet 1 compared to dogs fed Control diet.


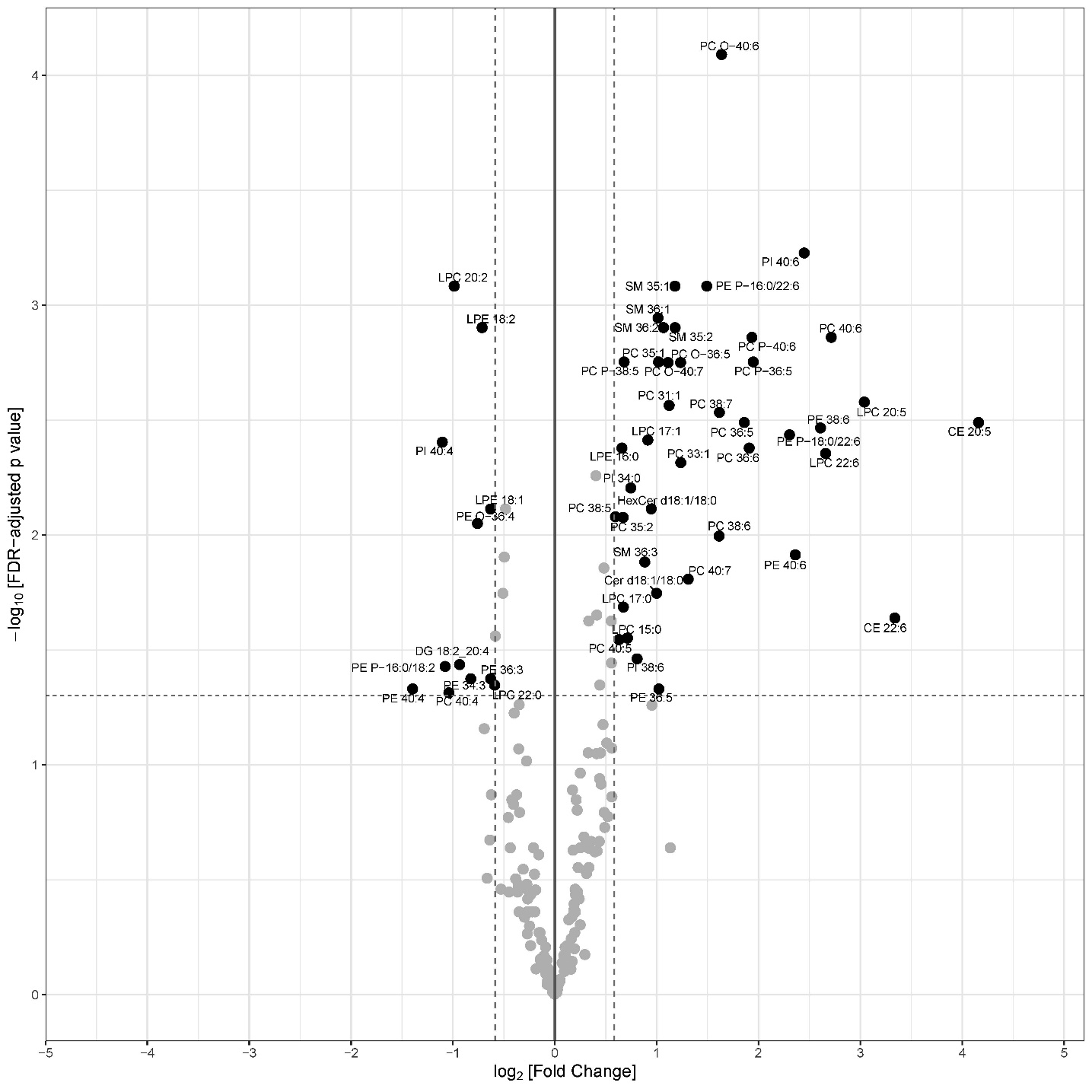


**Supplementary FigS5:** Volcano plot comparing all quantified lipid species, except of TGs, between Diet 2 and Control diet. Significantly changed lipid species (FDR-adjusted p value < 0.05, |FC| > 1.5, paired, two-tailed *t*-test) are indicated in red with the species name. The corresponding plot for TGs are shown in Supplementary FigS2. A positive FC means species has higher serum level in dogs fed Diet 2 compared to dogs fed Control diet.

**
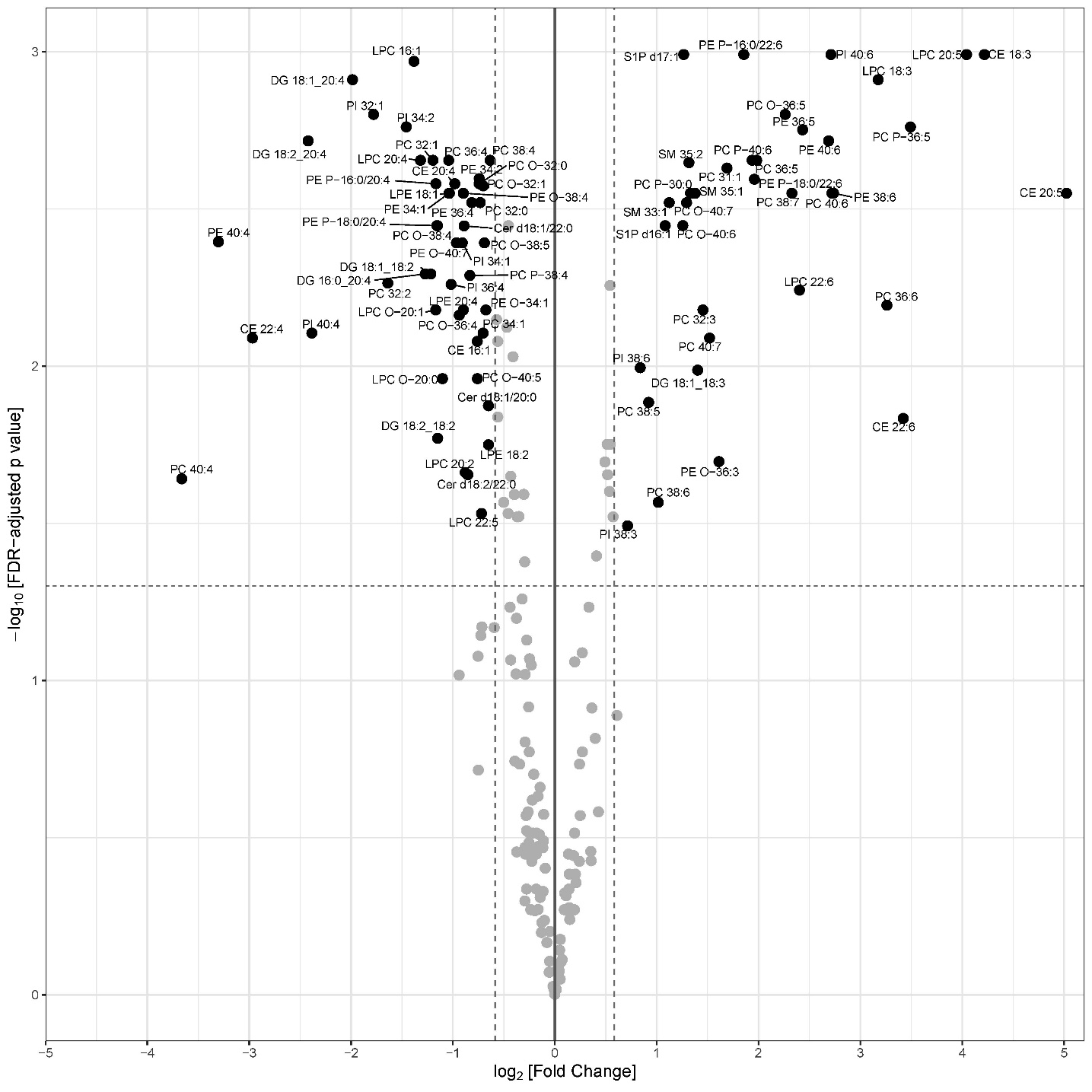
**

**Supplementary FigS6:** Volcano plot comparing all quantified lipid species, except of TGs, between Diet 2 and Diet 1. Significantly changed lipid species (FDR-adjusted p value < 0.05, |FC| > 1.5, unpaired, two-tailed Welch’s *t*-test) are indicated in red with the species name. The corresponding plot for TGs are shown in Supplementary FigS3. A positive FC means species has higher serum level in dogs fed Diet 2 compared to dogs fed Diet 1.

**
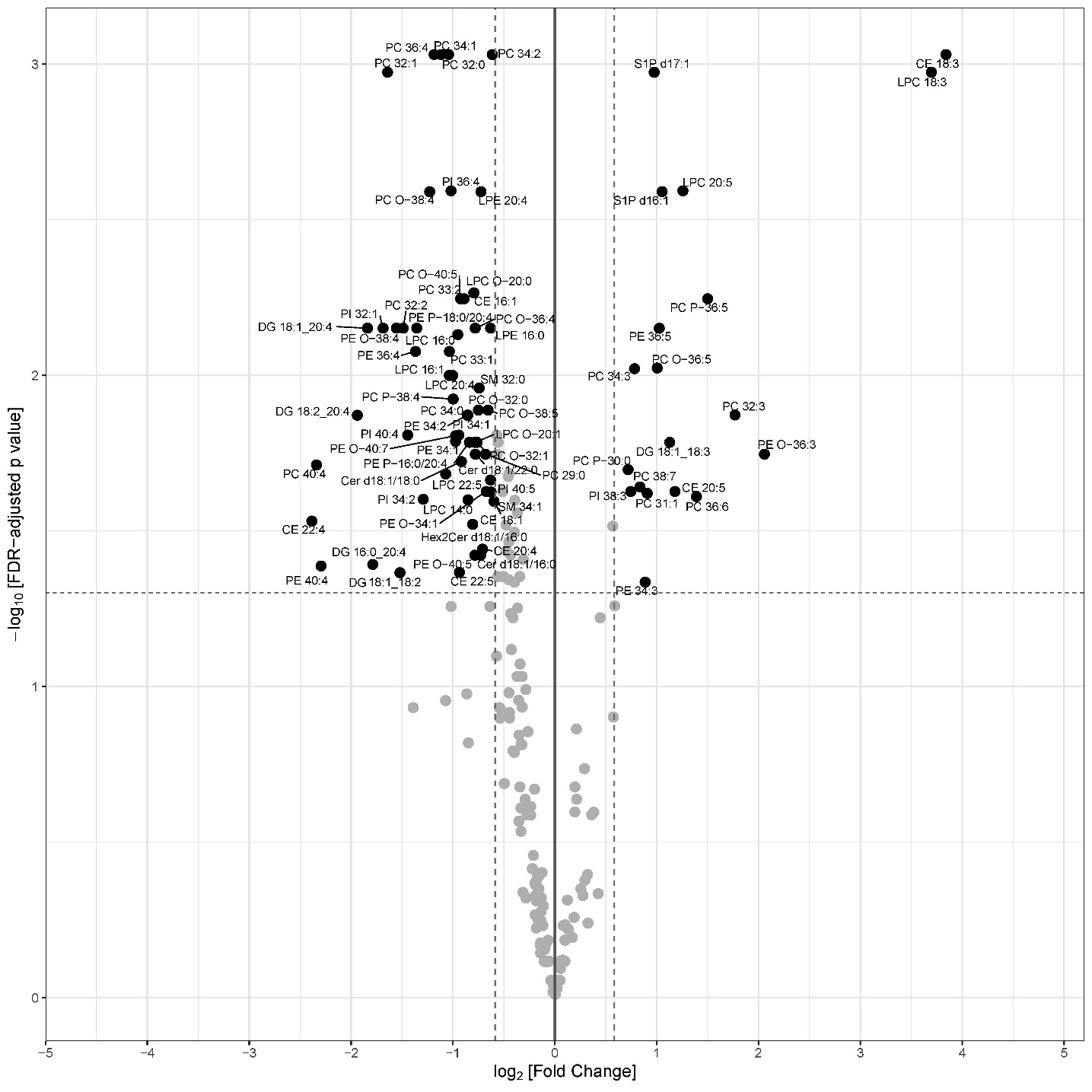
**

**Supplementary FigS7:** Dot plots of serum concentrations of all quantified lipid species. The stars above the bars indicate significance levels (* *P* < 0.05, ** *P* < 0.01, *** *P* < 0.001) from paired, two-tailed *t*-tests in case of Diet 1 *vs* Control diet and Diet 2 *vs* Control diet, and from unpaired, two-tailed Welch's *t*-tests in case of Diet 1 *vs* Diet 2. For exact *P* values please refer to Supplementary EXCEL table S4. The dashed grey lines depict the mean of the groups.


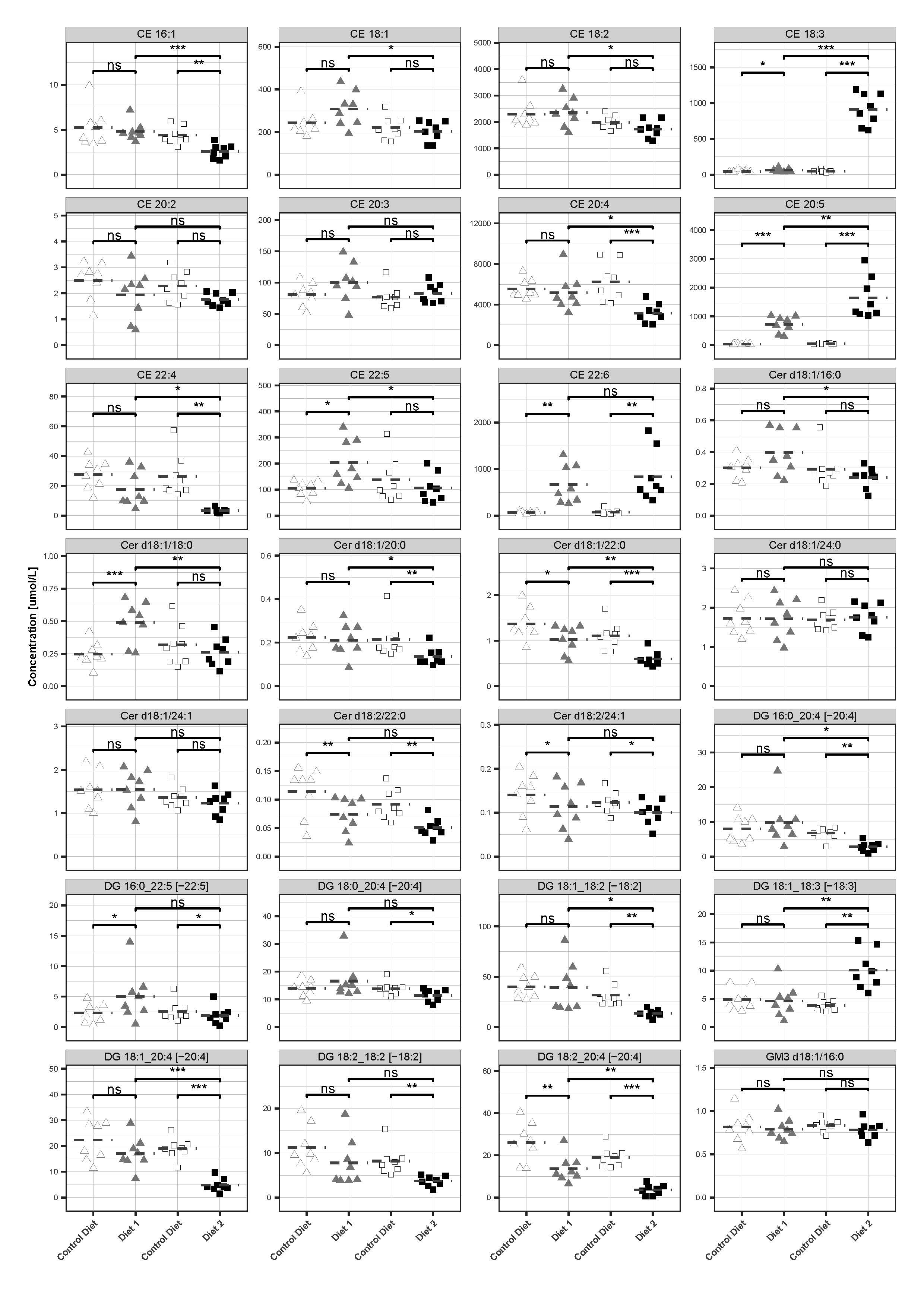


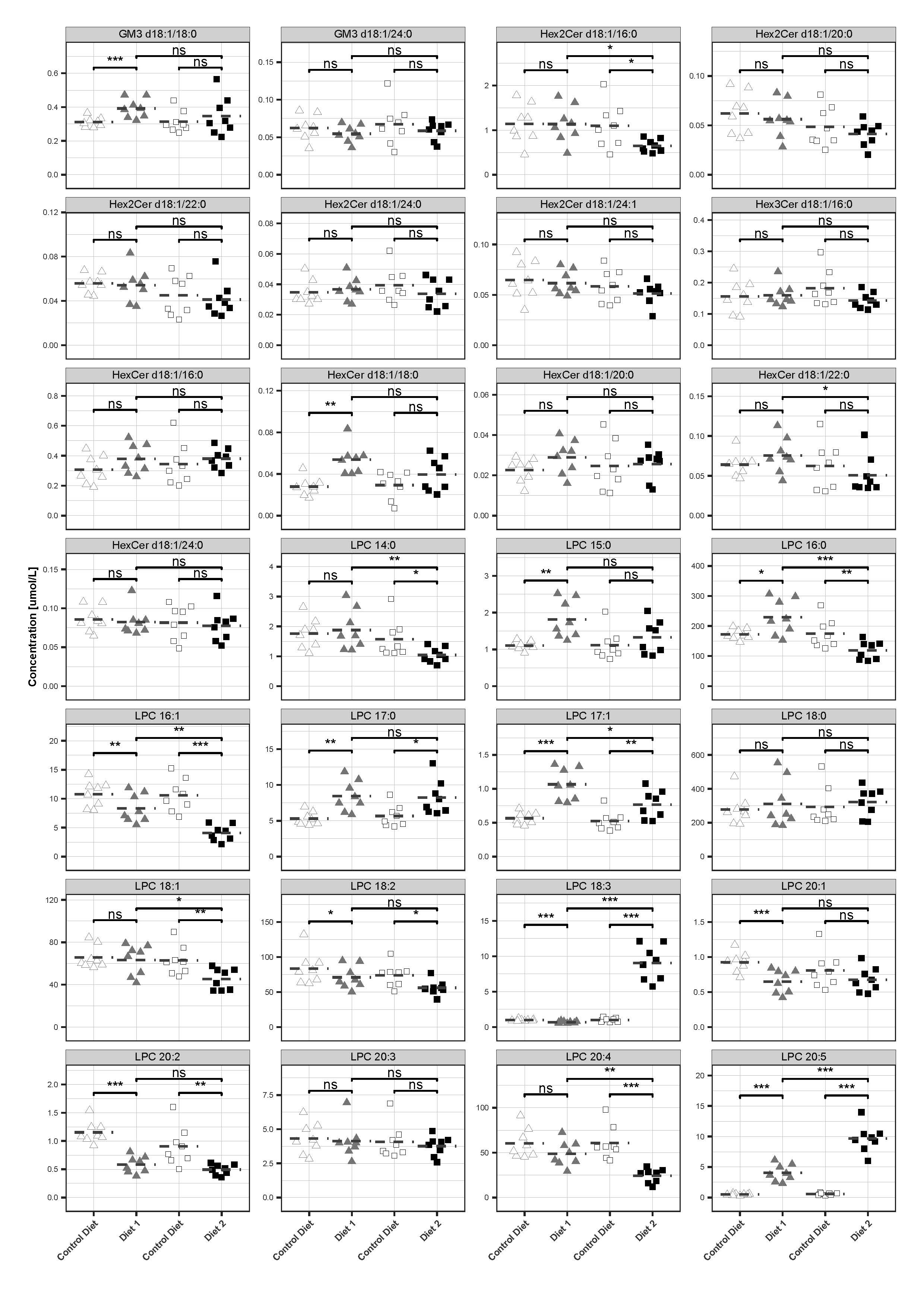


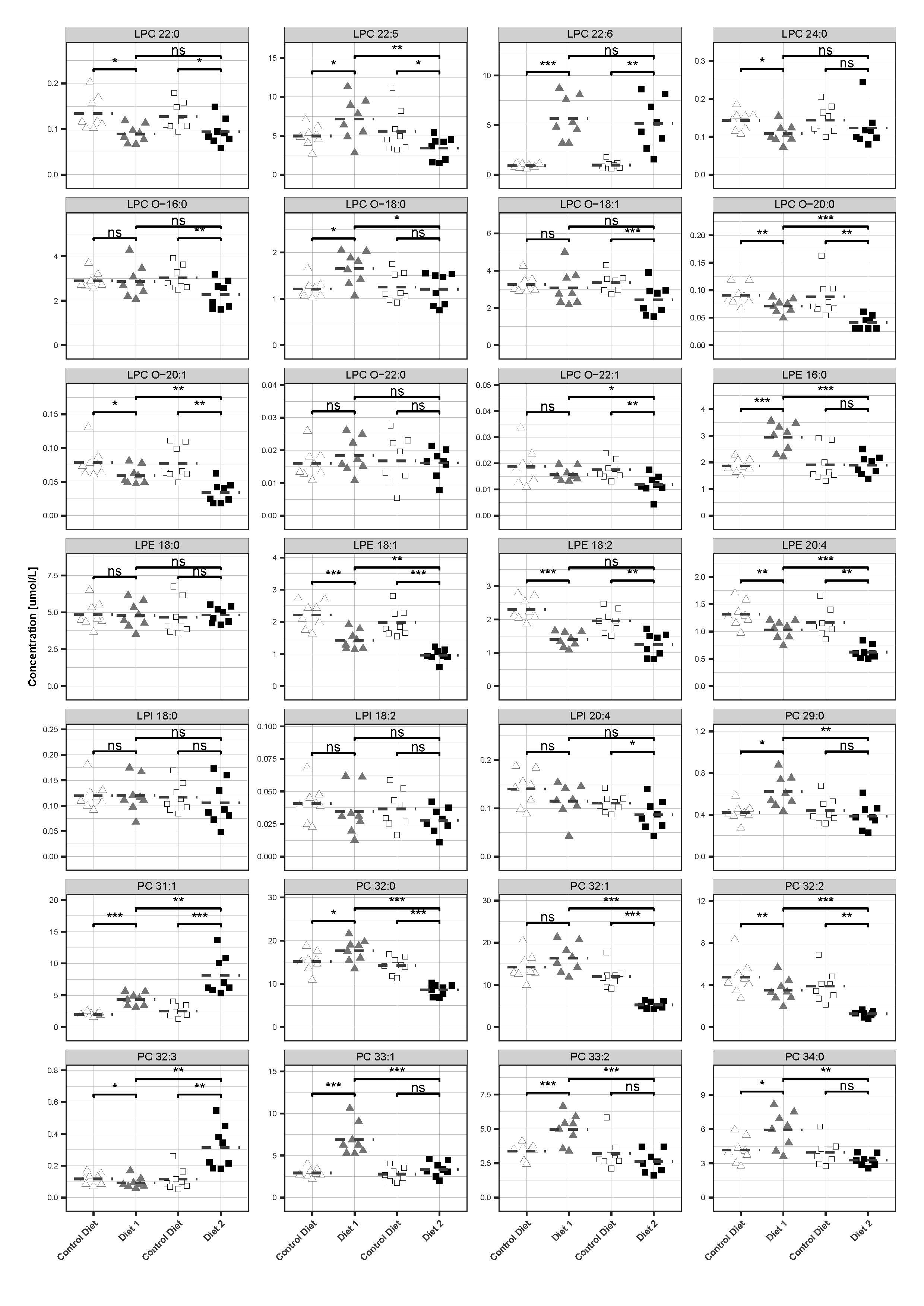


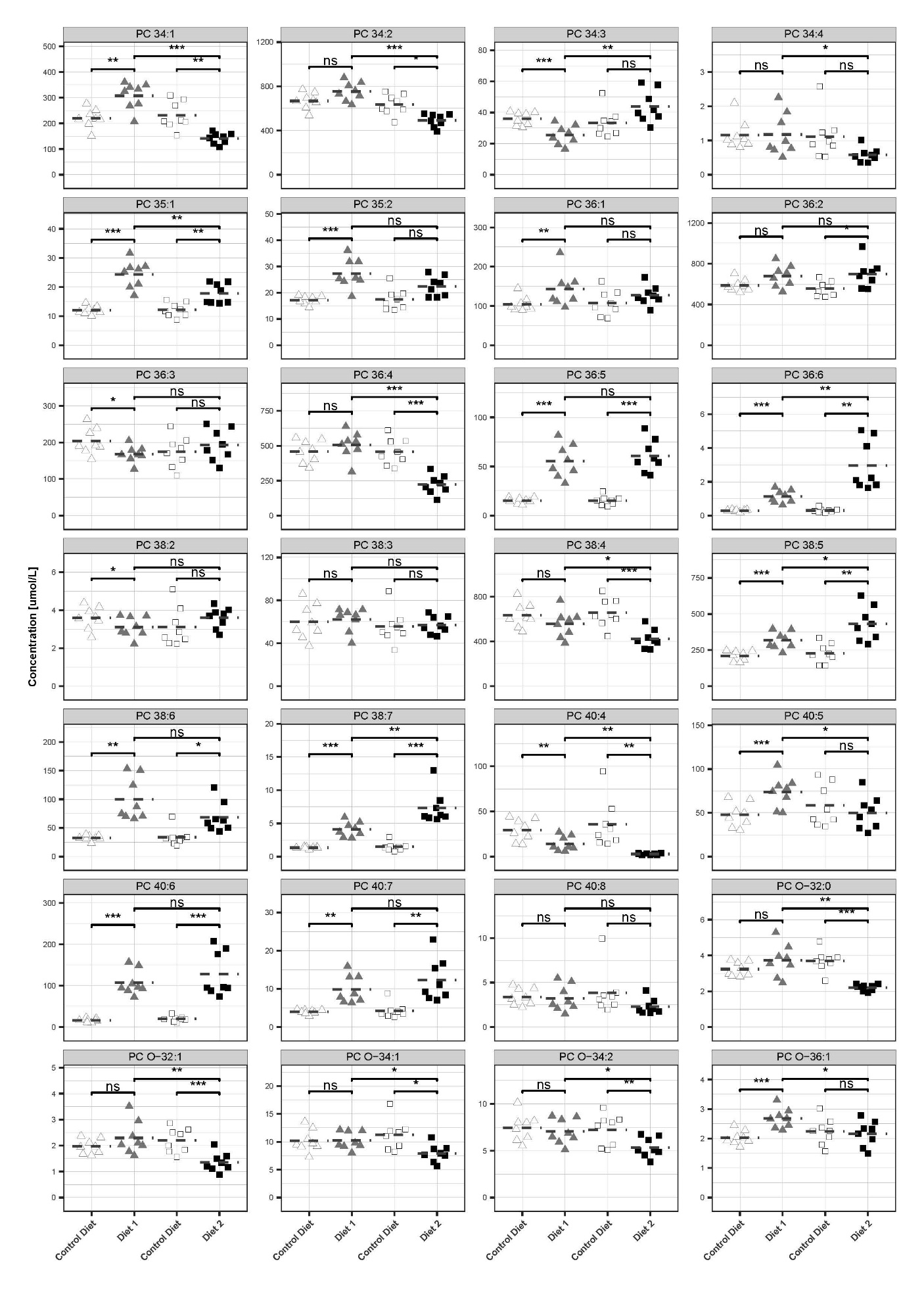


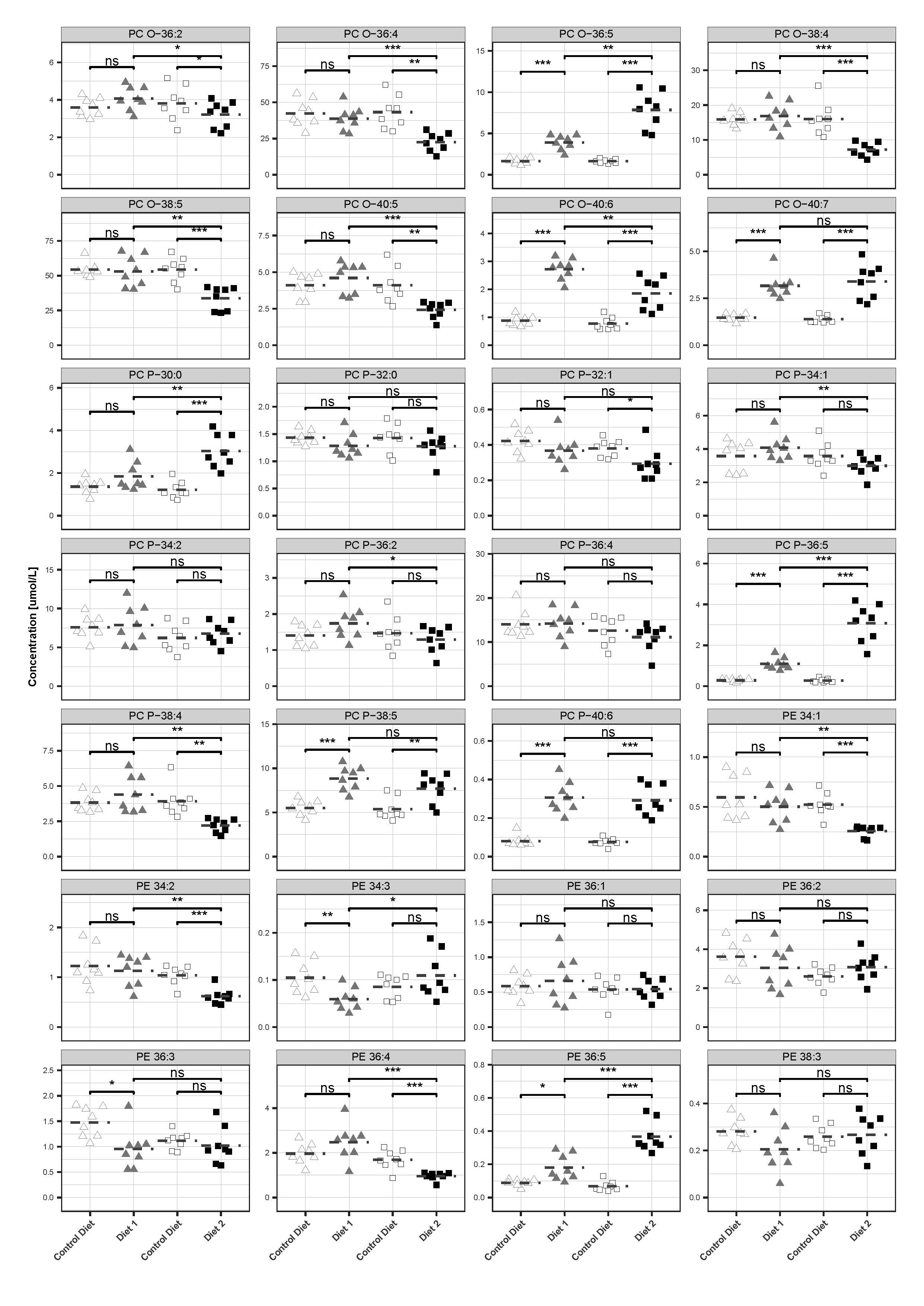


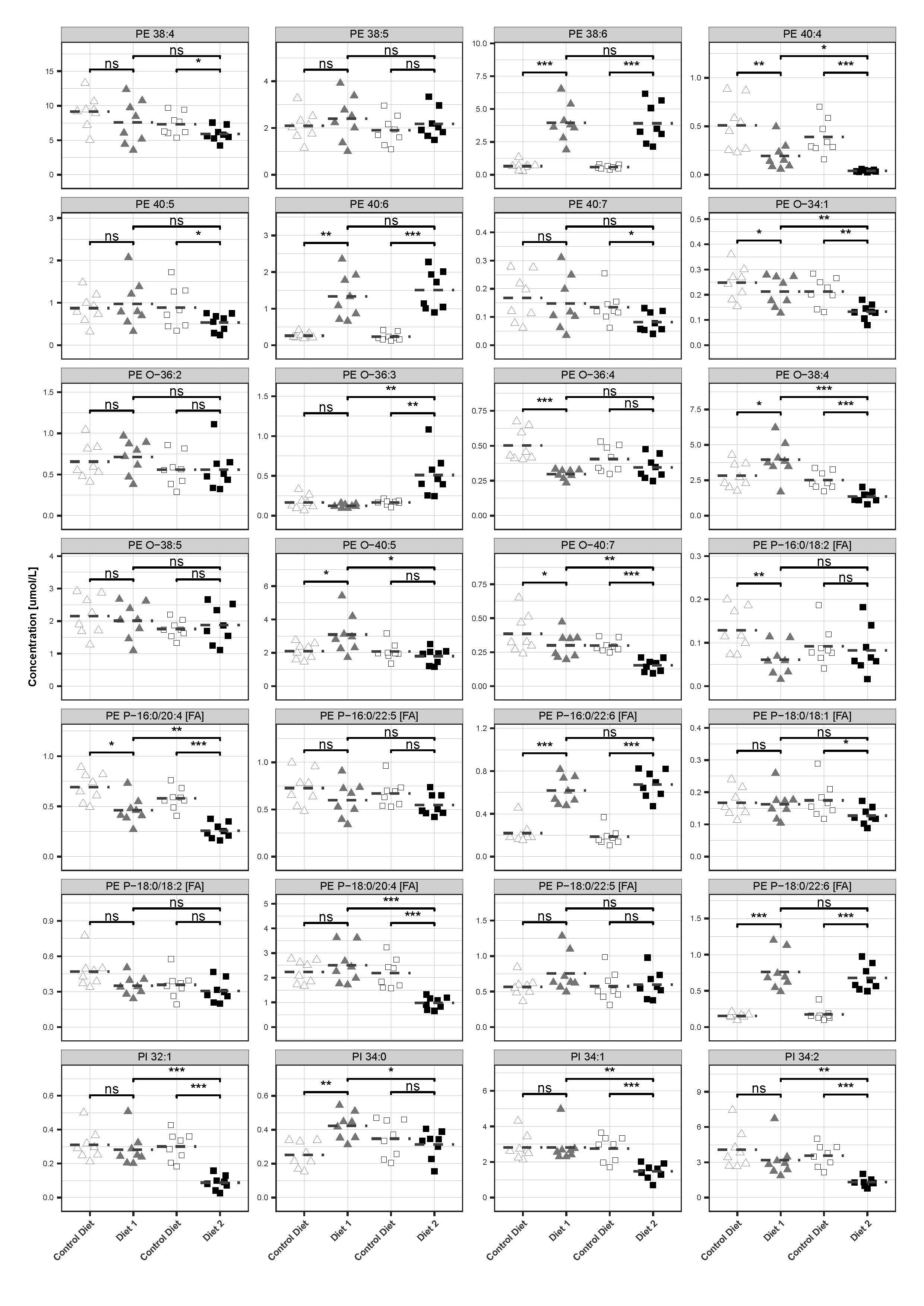


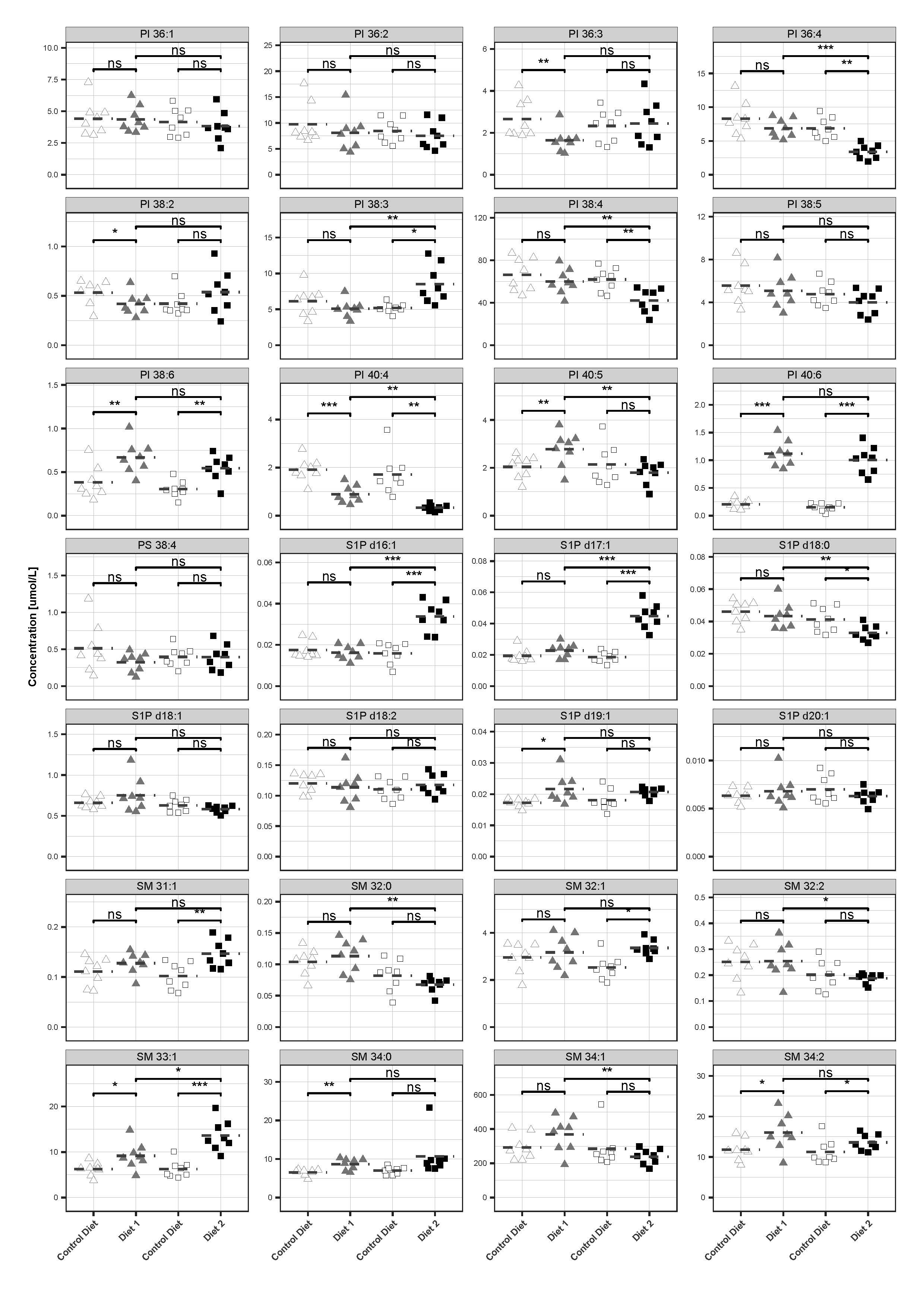


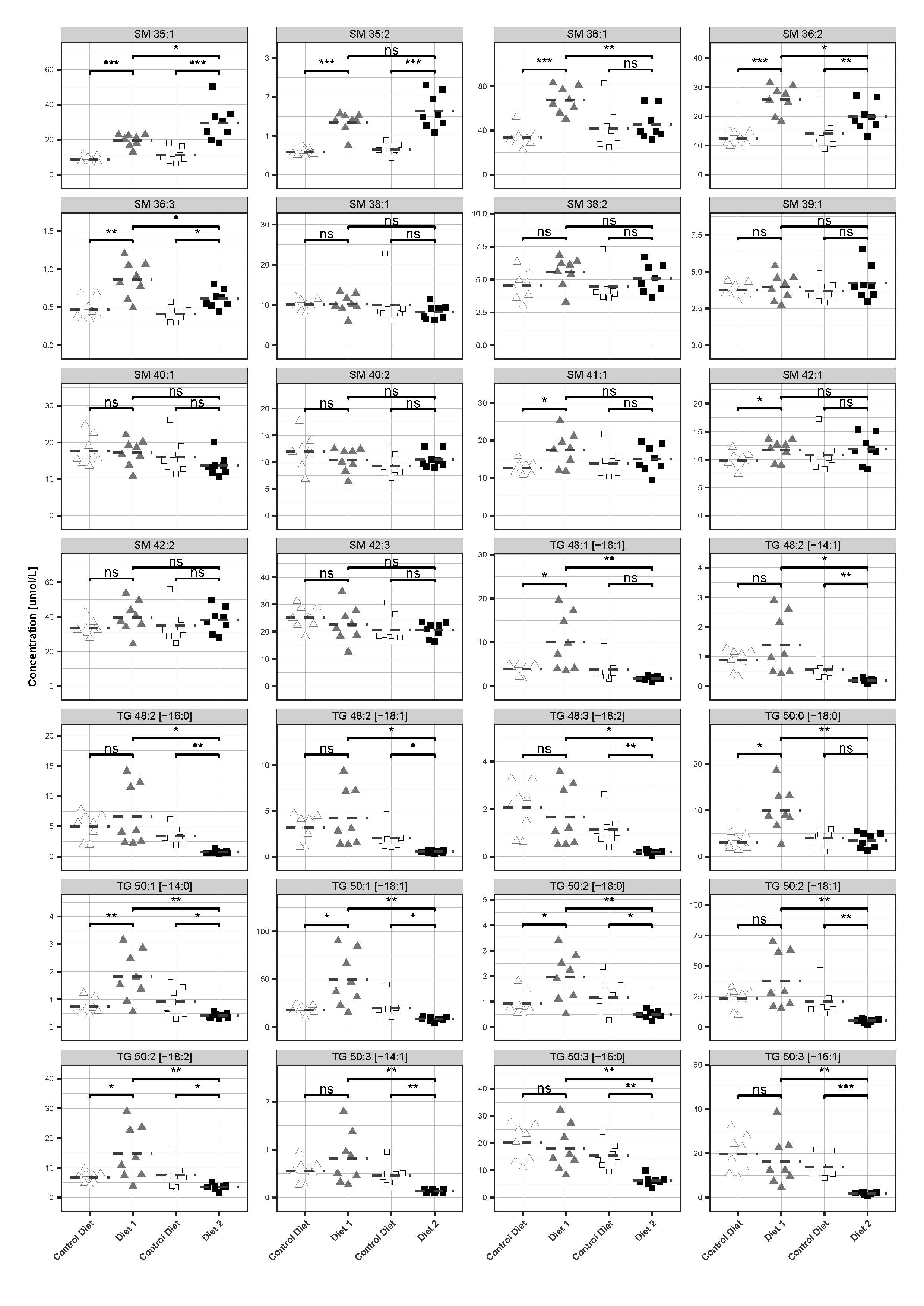


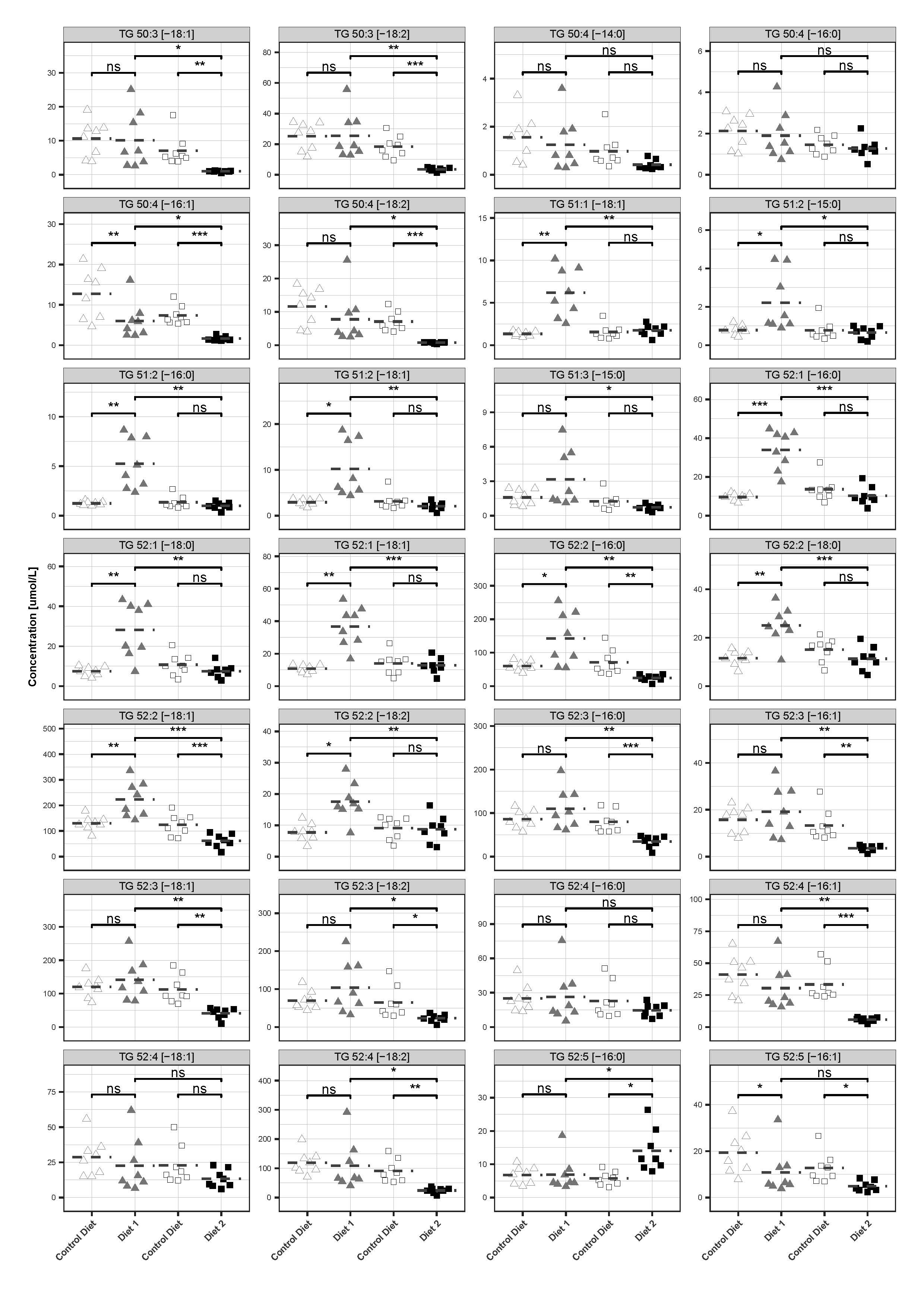


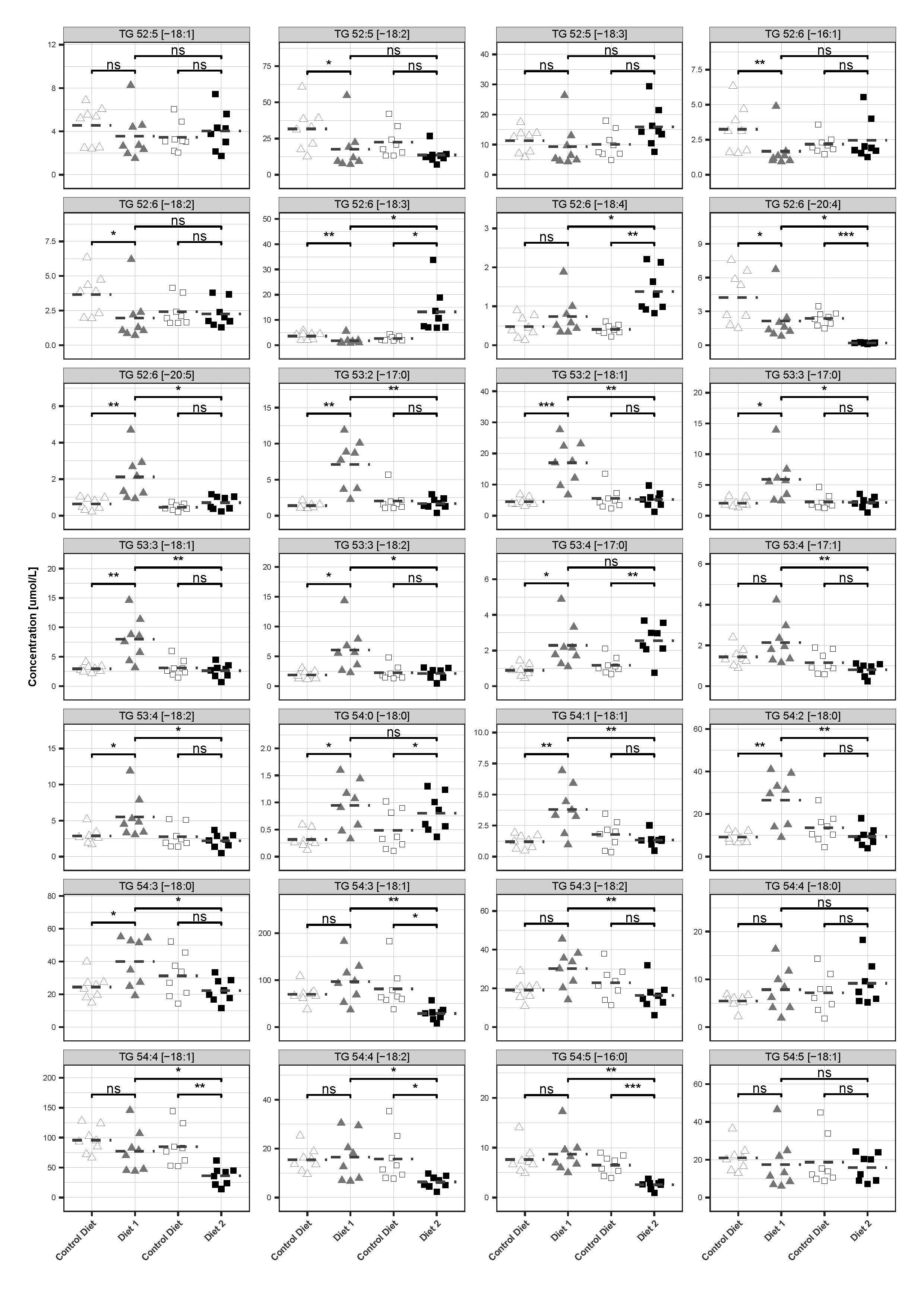


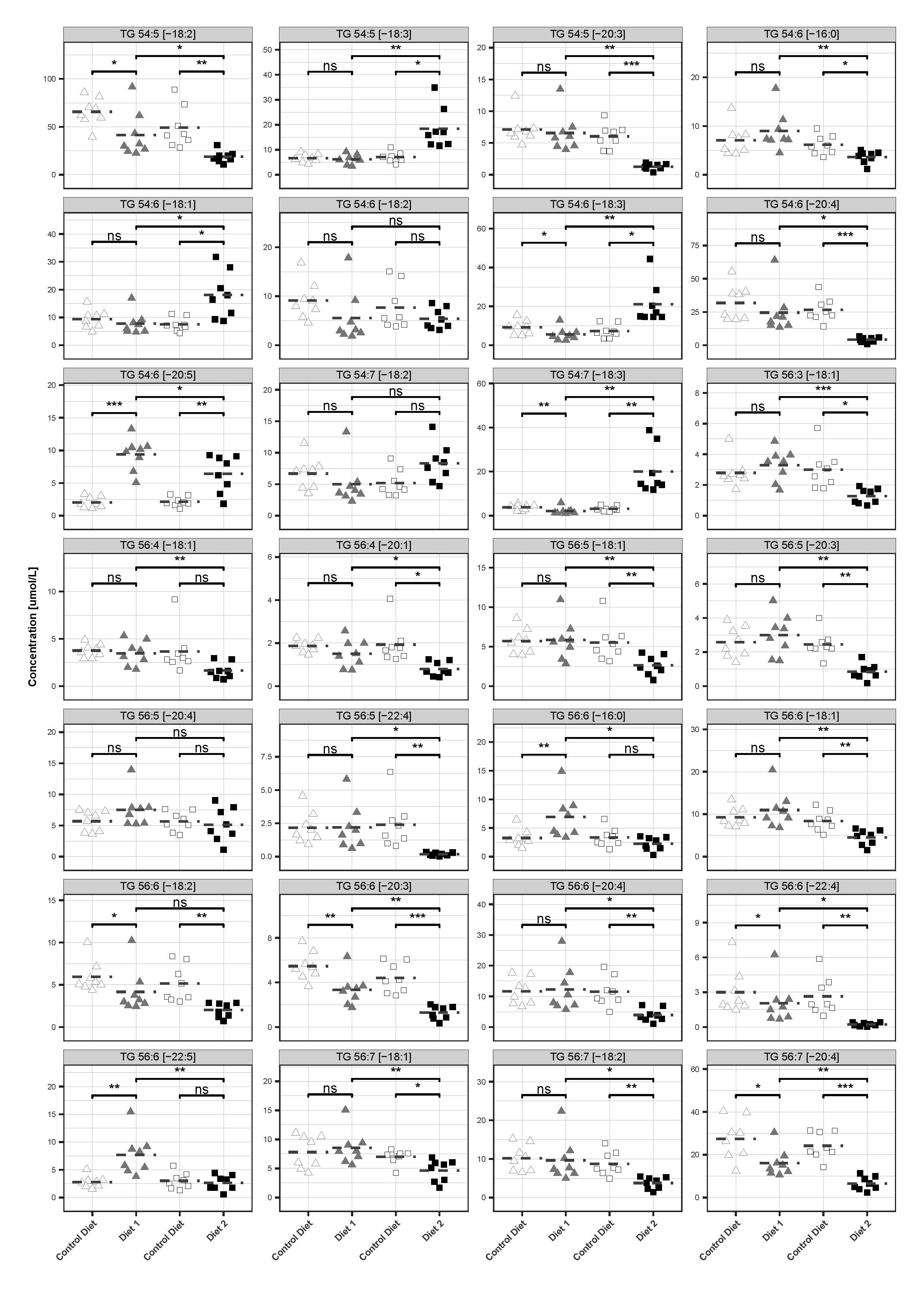


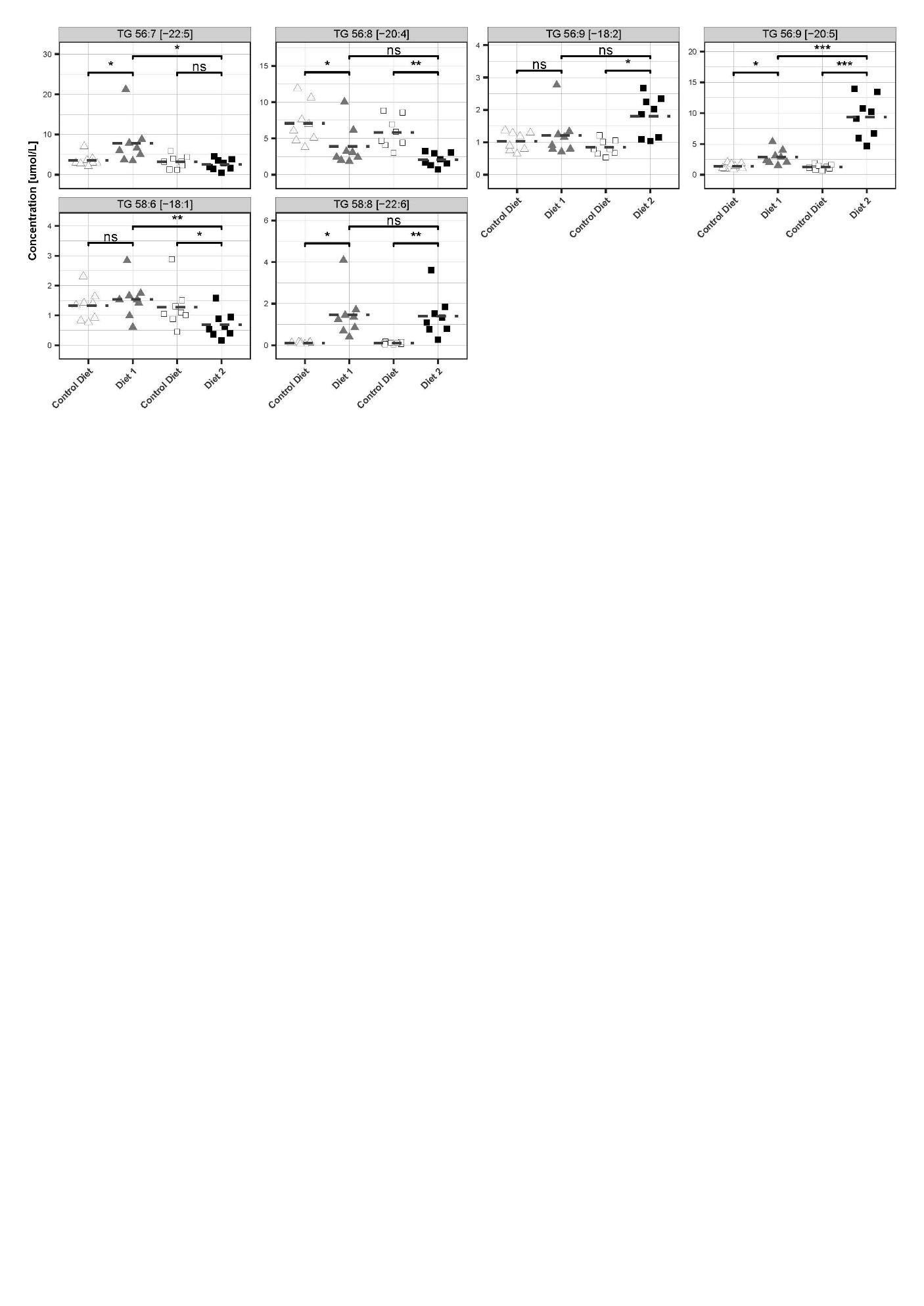

Supplement: Supplementary file 2 — Supplementary material 2 (DOCX 8704 kb) [file 11306_2019_1621_MOESM2_ESM.docx]
